# Is It Possible to Perform Triple Cross-Linking of Sodium Alginate? Development of Vaginal Sponges Containing Luliconazole Based on the Alginate/Gelatin/ɛ-Poly-L-Lysine/Zinc Matrix

**DOI:** 10.3390/pharmaceutics18101238

**Published:** 2026-09-29

**Authors:** Marta Szekalska, Monika Trofimiuk, Ewelina Piktel, Małgorzata Sidewicz, Eliza Wolska, Jolanta Magnuszewska, Paweł Misiak, Agnieszka Zofia Wilczewska, Katarzyna Winnicka

**Affiliations:** 1 Department of Pharmaceutical Technology, Medical University of Białystok, Mickiewicza 2C, 15-222 Białystok, Poland; monika.trofimiuk@umb.edu.pl (M.T.); katarzyna.winnicka@umb.edu.pl (K.W.); 2 Independent Laboratory of Nanomedicine, Medical University of Białystok, 15-222 Białystok, Poland; ewelina.piktel@umb.edu.pl; 3 Department of Medical Microbiology and Nanobiomedical Engineering, Medical University of Białystok, 15-222 Białystok, Poland; malgorzata.sidewicz@umb.edu.pl; 4 Department of Pharmaceutical Technology, Medical University of Gdańsk, Hallera 107, 80-416 Gdańsk, Poland; eliw@gumed.edu.pl; 5 Department of Polymers and Organic Synthesis, Faculty of Chemistry, University of Białystok, Ciołkowskiego 1K, 15-245 Białystok, Poland; jolmag@uwb.edu.pl (J.M.); p.misiak@uwb.edu.pl (P.M.); agawilczuwb@gmail.com (A.Z.W.)

**Keywords:** sodium alginate, gelatin, ε-poly-L-lysine, sponges, triple cross-linking, ionic cross-linking, zinc ions, luliconazole

## Abstract

**Background/Objectives:** Vaginal administration enables optimal drug concentrations at the site of administration, but its effectiveness is limited by factors such as the vagina’s self-cleansing mechanisms and fluctuations in physiological conditions. Due to the difficulties in treating and the frequent recurrences of vaginal candidiasis, new drug formulations are required to ensure more effective therapy and sustained release of antifungal agents. Mucoadhesive vaginal sponges are a promising drug formulation providing prolonged release of the active ingredient and its contact with the vaginal mucosa. The application of polymers and their modifications plays a key role in the development of drug forms with unique and desired technological parameters. **Methods:** Therefore, the focus of this research was the development of a three-stage sodium alginate cross-linking technique based on polyelectrolyte complexes with gelatin and ɛ-poly-L-lysine, and additional ionic cross-linking with zinc ions, to obtain mucoadhesive vaginal sponges with an antifungal agent—luliconazole. The prepared formulations were evaluated for their pharmaceutical, mechanical, and mucoadhesive properties. In addition, antifungal and anti-biofilm action against Candida strains and compatibility with the vaginal VK2 E6/E7 epithelial cell line of the prepared sponges were assessed. **Results**: It was noted that the triple cross-linking process increased the mechanical properties of the developed sponges, improved mucoadhesive properties to vaginal mucosa of drug-loaded formulations, and demonstrated a sustained LUL release profile. Moreover, prepared sponges possessed improved antifungal and anti-biofilm effects against *C. albicans*, *C. krusei*, and *C. parapsilosis*. The prepared triple cross-linked formulations were biocompatible with the human vaginal VK2 E6/E7 epithelial cell line, depending on the exposure time and applied concentration. **Conclusions**: Triple cross-linked alginate sponges containing luliconazole were successfully prepared based on polyelectrolyte complexes with gelatin and ɛ-poly-L-lysine and additional ionic cross-linking with zinc ions. The triple cross-linking process of alginate improved the mechanical, mucoadhesive, and antifungal properties of the developed formulations.

## 1. Introduction

Vaginal administration is an effective method for delivering therapeutic agents applied in the treatment of gynecological conditions. Owing to its specific anatomical structure and the high permeability of the mucous membrane, the vagina is an optimal site for the administration of topically acting medications. The vaginal route enables for a therapeutic effect to be achieved with lower doses, reducing systemic effects and the incidence of adverse reactions [1]. Vaginal preparations include liquid, semi-solid, or solid medicinal formulations that contain active ingredients, but may also serve as lubricants that do not contain medicinal substances. For treatment to be effective and comfortable, preparations should remain in place for a prolonged period, not cause irritation, be comfortable to apply, and not irritate the vaginal mucosa. Conventional vaginal preparations have several limitations, such as short retention time on the vaginal epithelium, leakage, and spotting, which cause discomfort in patients [2]. These issues stem from insufficient adhesion of the formulations to the vaginal mucosa, highlighting the need to develop innovative drug delivery systems. The implementation of mucoadhesive polymers enables a significant extension of the time the active ingredient remains on the mucosal surface [3].

Sponges are a solid dosage form characterized by a three-dimensional porous skeletal structure similar to that of traditional sponges. Owing to the presence of pores, they exhibit a high storage capacity and the ability to active ingredients controlled release, while also possessing favorable mechanical properties. Sponges with mucosal adhesion qualities are a novel drug formulation intended for local application to mucosal surfaces. One method of producing these sponges is freeze-drying, a process commonly used in the pharmaceutical industry [4].

Sodium alginate (ALG) is a polysaccharide classified as a natural polymer, distinguished by its high degree of biological safety, compatibility with human tissues, and capacity for biodegradation. This compound exhibits significant physicochemical properties, such as the ability to absorb water and swell, mucoadhesive qualities, and a propensity to form hydrogel structures. The most commonly applied technique for its gelation remains ionic cross-linking involving divalent metal cations (e.g., zinc ions), described in the literature as the “egg-box” model, which reflects the organization of polymer chains around the cross-linking ions. The structure of ALG might be intentionally modified both through cross-linking processes and as a result of the formation of polyelectrolyte complexes, which arise through electrostatic interactions with oppositely charged polymers. These complexes constitute a specific type of supramolecular system in which anionic and cationic macromolecules associate, leading to the formation of stable, ordered structures with altered rheological and mechanical properties. Given its anionic nature, ALG effectively forms polyelectrolyte complexes with positively charged compounds, such as ε-poly-L-lysine (PLL) and type A gelatin (GEL) [5,6].

In the absence of a polymer with ideal pharmaceutical properties, new strategies for modifying polymers are evaluated to enable the design of multifunctional drug delivery systems tailored to the specific site of administration and therapeutic requirements. A new approach involves strategies that combine various mechanisms of action within a single polymer structure. The application of more than one cross-linking mechanism might enable the simultaneous modification of a polymer’s mechanical properties, degree of swelling, stability, and mucoadhesive properties. An example of this approach is the study by Türkoğlu et al. in which the formation of alginate–chitosan polyelectrolyte complexes in the presence of ionic cross-linking agents, including sodium polyphosphate, sodium phosphomolybdate, ammonium molybdate, and sodium pyrophosphate, was analyzed. They observed that the simultaneous application of three cross-linking agents improved the stability of the resulting systems compared to the use of individual agents separately [5]. In addition, Zhang et al. developed a triple cross-linked alginate/dextran/PAMAM dendrimer hydrogel utilizing covalent, ionic, and Schiff base cross-linking simultaneously, resulting in an unique material with controlled mechanical and adhesive properties [6].

Therefore, the aim of this study was to evaluate the potential of triple cross-linked alginate hydrogels for the creation of polyelectrolyte complexes with GEL and PLL, as well as additional ionic cross-linking utilizing zinc (Zn^2+^) ions, to generate mucoadhesive vaginal sponges. Both processes are based on a similar mechanism involving interactions between groups or ions with opposite charges. However, these mechanisms differ in the nature of the interactions, the type of ionic donor, and the way the resulting network is organized. Polyelectrolyte complexes form as a result of electrostatic interactions between the negatively charged carboxylate groups of ALG and the positively charged amine groups of GEL and PLL. In the case of ionic cross-linking, divalent ions form coordination bridges with the carboxylate groups of surrounding ALG chains, leading to the formation of additional cross-linking sites and the formation of a spatially stable polymer network [7,8]. Triple cross-linking of polymers, utilizing different interaction mechanisms—ionic and electrostatic—simultaneously, leads to the formation of a well-organized and stable spatial structure with increased mechanical and chemical resistance. The application of this strategy enables the modification of rheological properties, swelling degree, and mucoadhesive properties, which is of significant importance in the design of materials with specific functionality. Furthermore, the complex network architecture facilitates the controlled release of active substances and influences biological properties, thereby increasing the application potential [8].

Luliconazole (LUL), an antifungal compound belonging to the imidazole derivative group, was selected as the model substance. Its antifungal activity is associated with the inhibition of 14α-lanosterol demethylase, which impairs the synthesis of ergosterol, a component of the fungal cell membrane [9]. In the initial part of this study, the composition and cross-linking process of hydrogel were developed. Following this analysis, specific hydrogel formulations were selected for the production of sponges via the freeze-drying technique. Next, a qualitative analysis was conducted, along with an evaluation of the effect of ALG modification on the properties of the developed sponges. Additionally, the resulting drug formulations were evaluated for antifungal activity and their effect on biofilm formation was examined using fungi of the genus Candida: *C. albicans*, *C. krusei*, and *C. parapsilosis*. The effect of triple cross-linking process on the compatibility of the formulations with the VK2 E6/E7 vaginal epithelial cell line was also examined.

## 2. Materials and Methods

Luliconazole (LUL) and ɛ-poly-L-lysine (PLL) were purchased from Kerui Biotechnology Co. LTD (Xi’an, China). Sodium alginate (ALG) from *Macrocystis pyrifera* characterized by M/G ratio of 1.56, 3.5 × 10^5^ Da molecular weight and medium viscosity amounting 2415 mPa·s for 1% solution at 25 °C, gelatin (GEL) from porcine skin type A, dimethyl sulfoxide (DMSO), calcium chloride, sodium dodecyl sulphate (SDS), and zinc sulphate heptahydrate (ZnSO_4_·7H_2_O) were acquired from Sigma Aldrich (St. Louis, MO, USA). Water was distilled utilizing the Milli-Q Reagent Water System (Billerica, MA, USA). Simulated vaginal fluid (SVF, pH = 4.2) contained 5 g glucose, 2 g lactic acid, 3.51 g natrium chloride, 1.40 g potassium hydroxide, 1.0 g acetic acid, 0.4 g urea, 0.222 g calcium hydroxide, 0.16 glycerol, and 0.018 g bovine albumin in 1 L of water [8]. Porcine vaginal mucosa was acquired from a local veterinary service facility. The collected tissue was divided into fragments and stored in a frozen state at −80 °C for a period not exceeding one month prior to experimental use. The described procedure did not require approval from the Local Ethical Committee for Animal Experiments. All other chemicals and reagents used throughout this study were of analytical grade. The stock cultures of *Candida albicans* ATCC^®^ 10231, *Candida krusei* ATCC^®^ 6528, and *Candida parapsilosis* ATCC^®^ 22019 and Sabouraud dextrose agar were acquired from Biomaxima (Lublin, Poland). Additionally, BD Difco™ Sabouraud Dextrose Broth, BD DIFCO™ Sabouraud Dextrose Agar media, and tryptic soy broth (TSB) growth medium were purchased from Argenta (Poznań, Poland). All other reagents were of analytical or HPLC grade. The commercial VK2 E6/E7 cell line, derived from vaginal epithelium, was obtained from American Type Culture Collection (ATCC, Manassas, VA, USA). Keratinocyte serum-free medium (KSF) and human recombinant EGF were from GIBCO (GIBCO, Fisher Scientific, Waltham, MA, USA). Phosphate-buffered saline solution (PBS) was obtained from PAN Biotech (Aidenbach, Germany), and thiazolyl blue tetrazolium bromide (MTT) was purchased from Sigma Aldrich (St. Louis, MO, USA). For the purpose of staining the cytoskeleton and nuclei, 36–38% solution of formaline (Chempur, Piekary Śląskie, Poland), ActinRed™ 555 ReadyProbes™ Reagent (Rhodamine phalloidin), and NucBlue™ Live ReadyProbes™ Reagent (Hoechst 33342) from Thermo Fisher Scientific (Thermo Fisher Scientific, Waltham, MA, USA), respectively, for cell stabilization were applied.

### 2.1. Cross-Linked Hydrogels Formation

Single Cross-linked Hydrogels Based on the ALG/GEL Polyelectrolyte Complexes Development

In the first stage, blends of ALG/GEL polymers, with concentrations based on the results of our previous studies, were created [10]. The 2% ALG solution was prepared by dissolving ALG in distilled water utilizing mechanical stirrer model RZR 2020 (Heidolph Instruments, Schabach, Germany). The 0.75% (*w*/*v*) GEL solution was prepared by adding GEL to distilled water and heating at 60 °C for 1 h while being stirred by the mechanical stirrer. Upon obtaining a homogeneous solution, it was cooled to 40 °C. The ALG/GEL complexes were prepared by mixing the component solutions at 40 °C.

Dual Cross-linked Hydrogels Based on the ALG/GEL/PLL Polyelectrolyte Complexes Development

To obtain the ALG/GEL polyelectrolyte complex, PLL was applied at a concentration determined on the basis of previous studies [11]. Afterwards, 5 mL 0.1% (*w*/*w*) PLL solution was dripped into the ALG/GEL mixture and subjected to mechanical stirring.

Triple Cross-linked Hydrogels Based on the Ionic Cross-linking of ALG/GEL/PLL Complexes with Zinc Ions

Then, 3% zinc sulphate heptahydrate (ZnSO_4_·7H_2_O) in different volume (2.5 mL, 5 mL, 10 mL, and 15 mL) was added into the ALG/GEL/PLL polyelectrolyte complex the mixture is stirred continuously to form uniform triple cross-linked hydrogels. LUL at 5.0% (*w*/*w*) content was dispersed evenly in the gel matrices. The control groups comprised non-cross-linked ALG solution and hydrogels without the active compound (preparations S0–S3).

#### Triple Cross-Linked Hydrogels Properties Analysis

The resulting formulations were evaluated 24 h after preparation with a rotational cone viscometer (RVDV-III Ultra, Brookfield Engineering Laboratories, Middleboro, MA, USA), with measurements taken at a temperature of 22 ± 1 °C with a CPA52Z cone (plate diameter 24 mm, cone angle 3°). The viscosity of the tested systems was specified at a fixed shear rate of 6.00 s^−1^. The rheological data were presented in the form of flow curves illustrating the dependence of viscosity on the shear rate gradient in the range of 2.00–10.00 s^−1^. Additionally, the hydrogels were subjected to pH analysis using a pH meter Orion 3 Star (Thermo Scientific, Waltham, MA, USA), enabling for the precise determination of the pH values of the tested samples. An analysis of the textural properties—including hardness, consistency, and cohesiveness of the samples—was conducted using a texture analyzer (Stable Micro System, Godalming, UK). These values were assessed with 10 mm diameter cylindrical probe, which was pressed into 30 g samples at a speed of 1 mm/s to a depth of 5 mm and then withdrawn.

### 2.2. Triple Cross-Linked Sponges Preparation

Based on the findings of the viscosity, pH, and texture analysis of triple cross-linked ALG/GEL/PLL/Zn^2+^ mixtures, the composition of the hydrogel which was selected for the sponges formulation is summarized in Table 1. Prepared hydrogels were distributed in 1.5 mL samples in plastic vials, frozen at −80° C for 24 h, and lyophilized by utilizing a freeze-dryer apparatus (Alpha 2-4 LSC Basic, Martin Christ, Osterode am Harz, Germany) under the conditions −20 °C for 24 h.

### 2.3. Triple Cross-Linked Sponges Assessment

#### 2.3.1. Evaluation of Sponges Morphology

Ten arbitrarily collected samples of sponges with varying formulations were subjected to analysis, during which their physical parameters— mass, length, and diameter—were assessed. In addition, the moisture content was determined using a thermogravimetric analyzer (Radwag WSP 50SX, Radom, Poland), which allows for the precise quantification of water in the tested formulations.

#### 2.3.2. Microscope Analysis

Surface analysis of the samples was performed utilizing a Keyence VK-X 3000 scanning confocal laser microscope (Keyence, Tokyo, Japan). Observations were performed in optical and laser-optical modes. A ×5 magnification objective was applied for image acquisition. In confocal mode, a laser with a wavelength of 404 nm (violet light) was employed, enabling the acquisition of high-resolution surface topography images and the analysis of the morphology of the examined samples. For analysis by scanning electron microscopy (SEM), sponges sprayed with a 2 nm gold layer were inspected by a Phenom Pro G5 microscope (Phenom World, Eindhoven, The Netherlands).

#### 2.3.3. Active Ingredient Amount

The LUL concentration was assessed by extracting samples from each sponge formulation in 15 mL of water, stirred for 1 h at 75 rpm in a heated water bath (37 ± 1 °C). Subsequently, 40 mL of methanol was introduced and mixed for 24 h. The LUL concentration was assessed at 300 nm using the High-Performance Liquid Chromatography (HPLC) technique with an Thermo Scientific Vanquish Duo (Thermo Scientific, Waltham, MA, USA) with a Poroshell^®^ 120 EC-C18 2.7 μM ODS 4.6 × 150 mm, 2.7 μm column (Agilent, Santa Clara, CA, USA). The eluent solvent was acetonitrile and water (80:20 *v/v*). LUL detection was determined at mobile phase flow rate 0.5 mL/min and wavelength of 300 nm. Active substance retention time was noted at 5.3 min [12]. The developed HPLC method for LUL determination was validated according to analytical validation requirements (ICH Q2(R2) Guideline Validation of Analytical Procedures) [13]. The procedures were validated in terms of system suitability testing, linearity, accuracy, and precision (repeatability and intermediate precision).

#### 2.3.4. Swelling Capacity

The sponges’ swelling capacity was measured in baskets of the USP apparatus designed for solubility testing. A 15 mL SVF solution with a pH of 4.2 at a temperature of 37 ± 1 °C was applied as medium. After specified time periods, the baskets were removed from the measuring beakers and drained thoroughly. The baskets containing the swollen sponges were then weighed and the swelling ratio (SR) was calculated using the following formula:

SR = (Ws − W_0_)/W_0_ (1)

where W_0_ is the sponge initial weight and W_S_ is the weight of swollen sponge [8].

#### 2.3.5. Mucoadhesiveness and Mechanical Properties

Mucoadhesiveness and mechanical qualities were determined by a TA.XT Plus texture analyzer (Stable Micro System, Godalming, UK). Mucoadhesive features, expressed as the detachment force (F_max_) and the work of adhesion (W_ad_), was measured with an A/MUC tool and porcine vaginal membrane was employed as a model to simulate the in vivo circumstances. In preliminary experiments, a pre-test speed of 0.5 mm/s was selected, a speed during the test of 0.1 mm/s, a contact time of 120 s, a speed after the test of 0.1 mm/s, and an applied force of 1 N. The mechanical specifications of all sponges’ formulations were presented as Young’s modulus, tensile strength, hardness, cohesiveness, and percent of elongation. The process variables included the pre-test, test, and post-test speed was 1 mm/s, strain 10%, distance 3 mm, and trigger force 0.001 N. The distance between the grips was 20 mm. Data acquisition and processing were undertaken employing Texture Exponent 32 software (version 5.0, Stable Microsystems, Godalming, UK).

#### 2.3.6. Ex Vivo Residence Time

**Figure 1:**
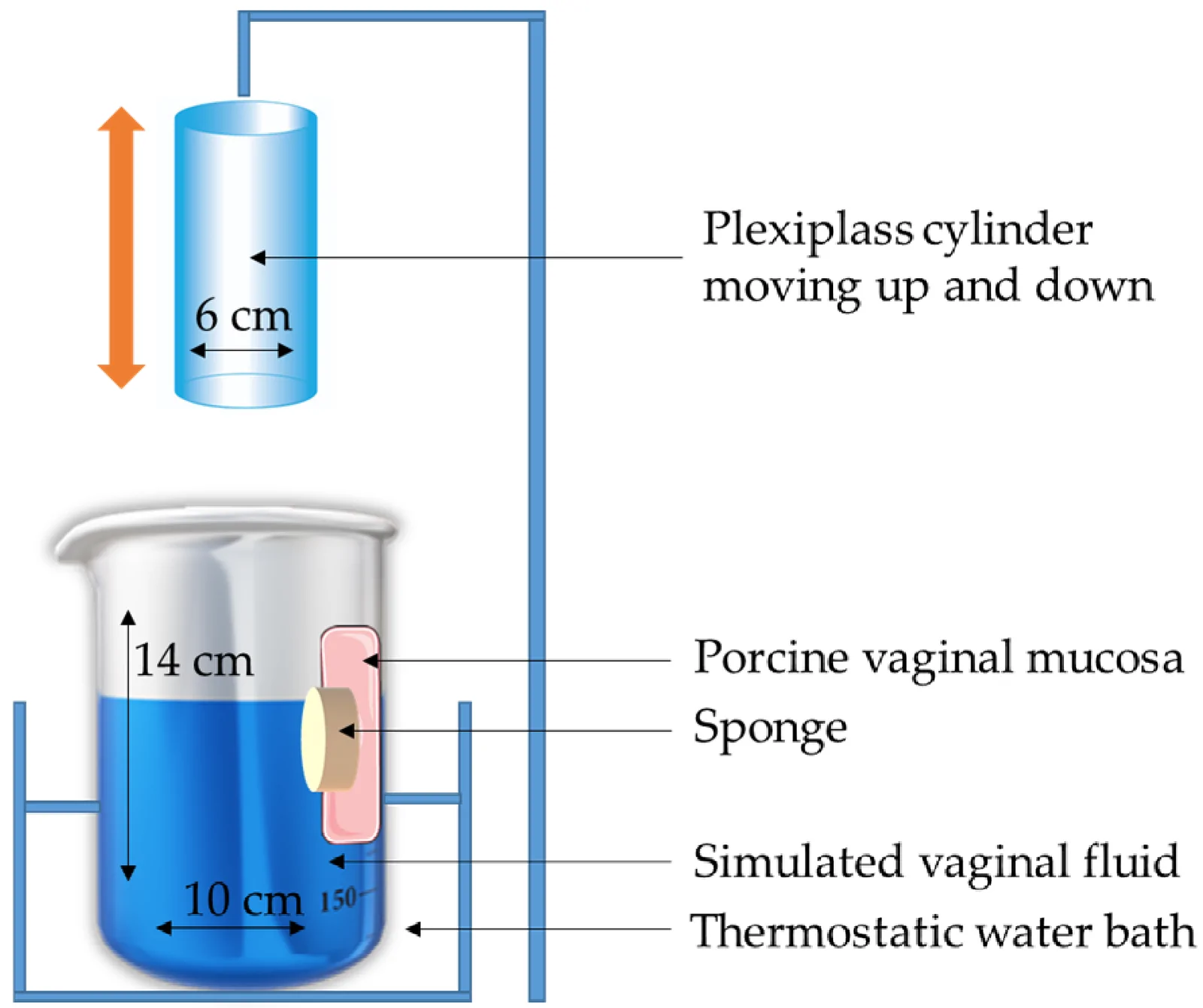
Scheme illustrating the analysis of sponges ex vivo residence time analysis with applying a modified pharmacopoeial disintegration apparatus (according to 14).

The retention time of the sponges was assessed according to the wash-off method, using a modified USP disintegration tester equipped with a 6 cm diameter, 280 g plexiglass cylinder that moved vertically up and down. The 5 × 3 cm porcine vaginal sections were attached to the inner surface of a beaker (Figure 1). The mucosa, along with the applied sponges, was then placed in beakers containing 700 mL of SVF (pH 4.2), held at a temperature of 37 ± 0.5 °C. The retention time was based on the moment of complete separation of the sponge from the mucosal surface [14].

#### 2.3.7. In Vitro LUL Release

In vitro release dissolution was estimated in the paddle apparatus (Erweka Dissolution Tester Type DT 600 HH, Heusenstamm, Germany). The sponges were put into containers with 500 mL of SVF buffer (pH 4.2) containing 1% SDS (to create “sink” conditions) and stirred at 75 rpm at 37 ± 1 °C. Sample collection was scheduled at 0.5, 1, 2, 3, and 4 h, and the solutions were then spectrophotometrically analyzed by a Genesys 10S UV-Vis instrument (Thermo Scientific, Waltham, MA, USA) at 300 nm. To elucidate the LUL release mechanism, the received results were analyzed according to zero-order kinetics, first-order kinetics, the Higuchi model, and the Korsmeyer–Peppas equation [15].

#### 2.3.8. Thermal Analysis

Thermal properties of the samples were investigated using thermogravimetric analysis (TGA) and differential scanning calorimetry (DSC). All measurements were performed using a Mettler Toledo Star^®^ instrument (Mettler Toledo. Star TGA/DSC unit, Columbus, OH, USA). Thermogravimetric analysis was carried out in the temperature range of 50–900 °C at a heating rate of 10 °C·min^−1^. Approximately 2.5 mg of each sample was placed in a ceramic measuring cell. Differential scanning calorimetry tests were conducted over a range of temperatures 25–300 °C, followed by cooling from 300 to 25 °C. The heating and cooling rates were determined to 10 °C·min^−1^ and −50 °C·min^−1^, respectively. Samples (2.5–3.0 mg) were sealed in aluminum pans using a hydraulic press, and an empty aluminum pan prepared under identical conditions was used as the reference.

#### 2.3.9. Attenuated Total Reflectance Fourier Transform Infrared Spectroscopy (ATR-FTIR)

All ATR-FTIR spectra were recorded in the wavenumber ranging from 4000 to 500 cm^−1^, employing a Thermo Scientific Nicolet 6700 FTIR spectrometer fitted with an ATR attachment. The spectra were acquired by summing 32 scans with a resolution of 4 cm^−1^ and were normalized against the reference spectrum.

#### 2.3.10. Antimicrobial Studies

##### Agar Diffusion Technique

The antimicrobial activity of the hydrogel preparations was evaluated in compliance with the guidelines of the Clinical and Laboratory Standards Institute (CLSI) [16]. Candidal fungus cells were applied in the study: *Candida albicans* ATCC^®^ 10231, *Candida krusei* ATCC^®^ 6528, and *Candida parapsilosis* ATCC^®^ 22019. The optical density of the inoculum (0.5 on the McFarland scale (5 × 10^6^ CFU/mL) in a sterile 0.9% NaCl solution) was assessed by suspension turbidity detector (DEN-1B densitometer, Biosan, Riga, Latvia). Developed sponges were put in 5 mm-diameter wells of agar plates. As control, a LUL in DMSO (50 μL) solution was applied. After incubation at 37 ± 0.1 °C for 24 and 48 h, the growth inhibition zones were determined by a caliper (Mitutoyo, Kawasaki, Japan).

##### Analysis of Biofilm Formation Against Candida Strains

For the purposes of biofilm formation analysis, the freshly prepared formulations were cut and suspended in 5 mL of cell culture medium Keratinocyte serum-free (KSF) supplemented with 0.1 ng/mL human recombinant EGF, 0.05 mg/mL bovine pituitary extract, and calcium chloride at the final concentration of 0.4 mM. After suspension, the mixture was left at 37 °C for 24 h, after which the insoluble fraction was centrifuged (2500 rpm, 10 min, room temperature) and the resulting supernatant was diluted in a fresh portion of medium to final concentrations of 1%, 10%, and 25%. In experiments conducted in the presence of SVF, the suspension was supplemented with 50% SVF, yielding the final formulation concentrations as described. LUL suspended directly in cell culture medium and maintained under the same conditions as the formulations was used as a control. The antimicrobial properties of the tested formulations were assessed by evaluating their ability to inhibit fungal biofilm formation. *C. albicans*, *C. parapsilosis*, and *C. krusei* cells in the logarithmic growth phase were suspended in tryptic soy broth (TSB) growth medium at approximately 5 × 10^7^ CFU/mL and incubated in the presence of the tested formulations for 24 h at 37 °C. Biofilm formation by fungal strains was assessed by a fluorometric method using resazurin (at a final concentration of 200 µg/mL, read at wavelengths of 520/590 nm) as a survival indicator. The data were collected at excitation/emission wavelengths of 520/590 nm by microplate reader device Varioskan LUX (Thermo Fisher Scientific, Waltham, MA, USA).

#### 2.3.11. Biocompatibility Sponges Evaluation

The VK2 E6/E7 cell line, derived from vaginal epithelium, was applied for the cell culture experiments. The cells were cultured at 37 °C, 5% CO_2_ in the presence of KSF medium indicated above until 80% confluence was reached. At 48 h prior to the experiment, the VK2 E6/E7 cells were seeded at a density of 15,000 cells per well, and the tested formulations prepared as described in the Section Analysis of Biofilm Formation Against Candida Strains were added. The suspension was supplemented with 50% SVF, yielding the final formulation concentrations as described. After 24 h at 37 °C, cell viability was assessed using the MTT assay. For this purpose, the cells were washed with PBS, followed by the addition of MTT solution (1 mg/mL) and incubation for 4 h and 24 h. After dissolving the insoluble formazan in sterile DMSO, absorbance was measured at a wavelength of 540 nm. As an additional test of cell viability, fluorescence imaging was performed on fixed cells (3.7% buffered formalin for 15 min, room temperature) stained for the cytoskeleton and cell nuclei using ActinRed™ 555 ReadyProbes™ Reagent (Rhodamine phalloidin) and NucBlue™ Live ReadyProbes™ Reagent (Hoechst 33342), respectively.

### 2.4. Statistical Analysis

The experimental data were subjected to analysis utilizing Statistica 12.0 software (StatSoft, Tulsa, OK, USA). Quantity variables were introduced as the mean and standard deviation. The statistical analysis was performed using one-way analysis of variance (ANOVA) and the Kruskal–Wallis test, with a significance level of *p* < 0.05.

## 3. Results and Discussion

ALG is a polysaccharide with unique gelling characteristics resulting from its ability to form cross-linked structures through both chemical and physicochemical interactions. The classic and most commonly implemented mechanism for forming alginate hydrogels remains “egg-box” ionic cross-linking, also referred to as ionotropic gelation, which involves the coordination of divalent or trivalent cations with guluronic acid blocks in the polymer chain. This process leads to the formation of a stable, spatially ordered, three-dimensional hydrogel matrix with high structural integrity. The most commonly applied cross-linking agents are calcium ions (Ca^2+^), but their utilization is associated with limitations, such as a rapid, difficult to control gelation process and the potential instability of the resulting structures [8]. Consequently, alternative cross-linking ions have emerged, particularly zinc cations (Zn^2+^), which exhibit a stronger affinity for ALG segments, enabling the formation of a more extensive and uniform three-dimensional network. This results not only in improved mechanical properties, but also in a prolonged release kinetics of active substances. An additional advantage of Zn^2+^ ions is their antimicrobial properties, including antifungal activity, which significantly increases the application potential of the resulting materials [17].

In addition to conventional ionic cross-linking, ALG exhibits the ability to form complex hydrogel systems through the formation of polyelectrolyte complexes. This phenomenon is based on electrostatic interactions between the anionic ALG carboxylate groups and the cationic centers of other polymers, such as PLL or GEL. The resulting systems are distinguished by improved mechanical, mucoadhesive, and swelling properties, as well as the ability to modify the release profile of active substances [18]. GEL, a natural high molecular weight polypeptide, obtained through the partial hydrolysis of collagen, is widely applied in pharmaceutical and biomaterials technology. It is capable of forming gels and exhibits film-forming properties. When ALG is combined with GEL, it has the potential to form systems with synergistic properties that find applications in, among others, controlled release systems for active substances, tissue engineering, and the design of modern drug delivery systems [19]. PLL is a cationic peptide with documented biocompatibility, biodegradability, and a broad spectrum of antimicrobial activity, including bacteriostatic and antifungal effects. Its presence in polymer systems might further impart protective properties to materials and enhance their functionality in biomedical applications [20].

**Figure 2:**
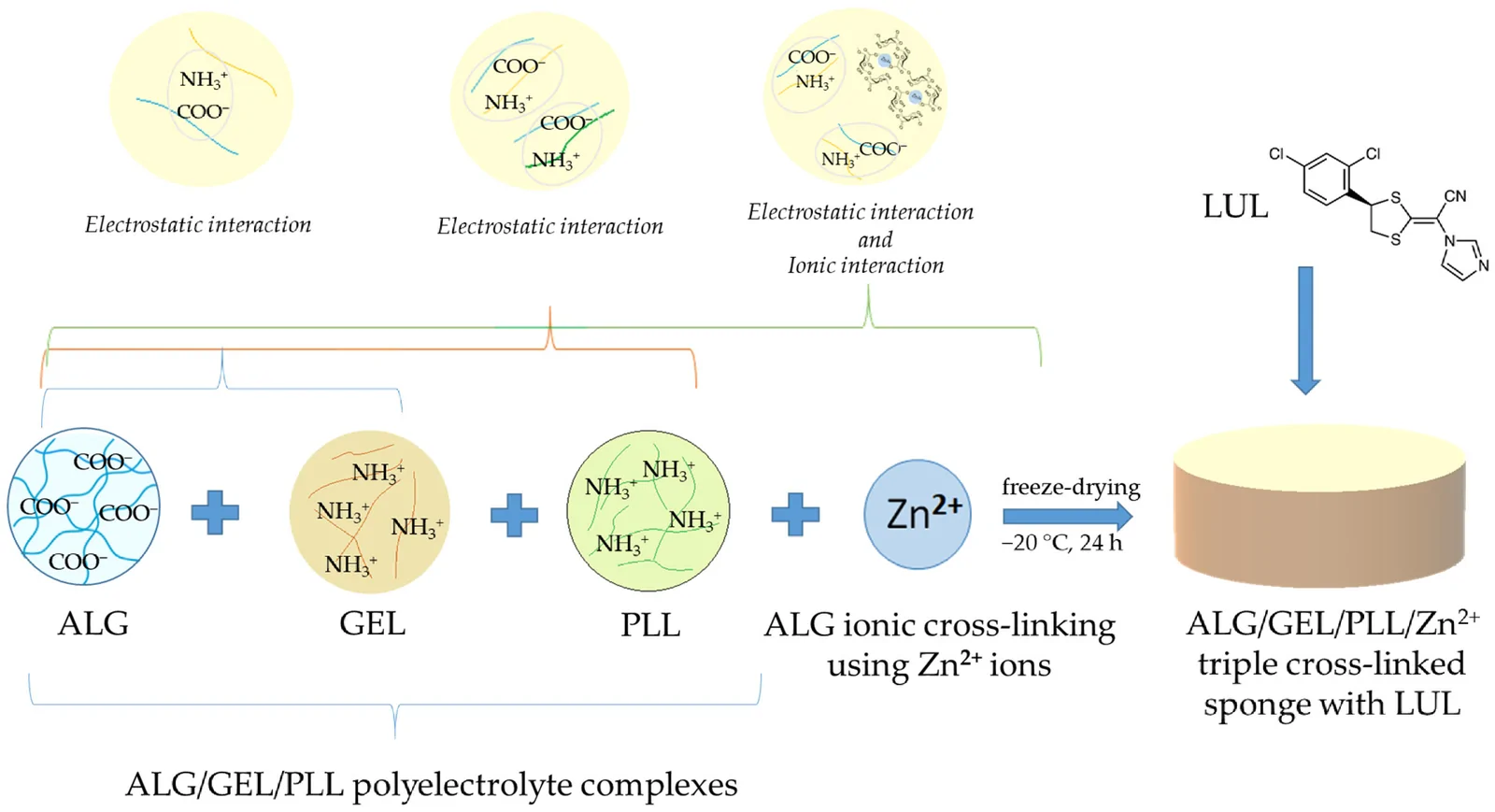
Concept of ALG triple cross-linking by GEL, PLL, and Zn^2+^ ions.

This study involved an innovative approach focused on developing a technique for obtaining ALG hydrogels through the triple cross-linking process with two methods: cross-linking via the formation of polyelectrolyte complexes with type A GEL and PLL, and ionic cross-linking using Zn^2+^ ions (Figure 2). Concentrations of GEL and PLL were selected for the preparation of polyelectrolyte complexes based on our previous research results [10,21]. For ionic cross-linking, a 3% zinc sulphate heptahydrate solution was selected, based on a series of experimental studies. Triple cross-linked gels were prepared with different volumes of the 3% Zn^2+^ ion solution, and the resulting hydrogels were analyzed for pH, viscosity, and rheological and mechanical properties.

### 3.1. Hydrogel Assessment

**Figure 3:**
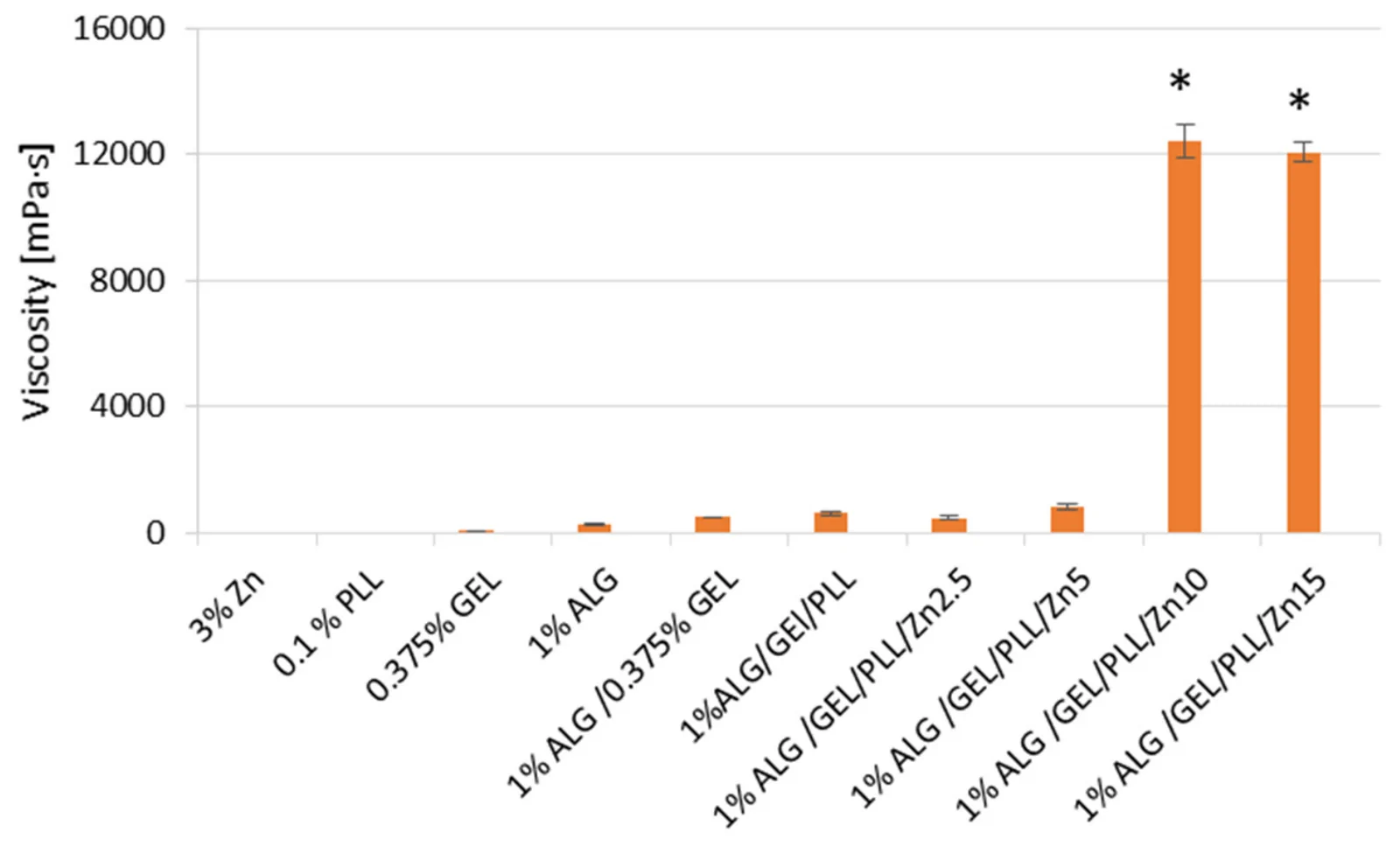
Viscosity values of ALG/GEL/PLL mixtures cross-linked with different volumes of 3% Zn^2+^ ions solution and individual ingredients (mean ± SD, *n* = 3, * significant statistical difference (*p* < 0.05) versus correspondent value of 1% ALG solution).

The hydrogel’s viscosity is influenced by multiple factors, including type of polymer, the type and concentration of the cross-linking agent, and the cross-linking process. Increasing the concentration of the formulation components leads to a denser three-dimensional network and an increase in the number of interactions between polymer chains. As a result, stability improves, but at the same time flow resistance increases, which complicates technological processes such as dosing and the filling of containers. Higher viscosity also causes a delay in the diffusion of active ingredients, which results in their slower, controlled release from the gel matrix. Furthermore, an increased polymer content and higher concentration of cross-linking agents might significantly influence the mechanical properties of the hydrogel. Both too low and too high a viscosity of the system are undesirable, as each leads to a deterioration in its performance and properties [22,23]. Therefore, it is crucial to precisely optimize the concentration of the polymer and cross-linking agents to achieve a balance between the hydrogel’s physicochemical stability, viscosity, and functionality, which will significantly influence the properties of the resulting sponges. Data derived from the viscosity evaluation of all prepared hydrogel formulations revealed that cross-linking promoted an increase in the investigated parameter (Figure 3, Table 2). The graph shown in Figure 2 indicates that the cross-linked hydrogels exhibited higher viscosity compared to the solutions of the unmodified components. ALG cross-linking with GEL practically doubled the viscosity, while dual cross-linking (ALG/GEL/PLL formulation) increased it threefold. It was demonstrated that the triple cross-linking process using 2.5 mL of a 3% Zn^2+^ ion solution resulted in a decrease in viscosity compared to the dual cross-linked hydrogel with GEL and PLL. In contrast, the application of 10 and 15 mL of the 3% Zn^2+^ ions solution resulted in an almost sixfold increase in hydrogel viscosity compared to the uncross-linked hydrogel. It was shown that all cross-linked hydrogel mixtures were uniformly homogeneous and demonstrated no indication of phase separation.

The formation of the polyelectrolyte complexes was considered in relation to the ionization state of the individual polymers and the pH of the formulations. ALG is negatively charged at the investigated pH range, as its carboxyl groups have pKa values of approximately 3.4–4.4, whereas GEL type A is positively charged at pH values below its isoelectric point (approximately 7–9). PLL remains protonated within the investigated pH range due to the high pKa of its ε-amino groups (approximately 10–11). The pH values of the formulations measured after mixing all components and prior to lyophilization were ranged from 7.48 ± 0.03 to 9.10 ± 0.02 (Table 2). It was found that the addition of GEL and PLL reduced the pH of the hydrogels. These data confirm that ALG and PLL retain the appropriate opposite charge characteristics for electrostatic interactions, while the degree of protonation of GEL type A may decrease as the pH approaches or exceeds its isoelectric point. In contrast, the addition of Zn^2+^ ions caused the pH of the hydrogels to decline to a neutral pH. In addition, the pH values decreased as the volume of the cross-linking solution applied increased.

**Figure 4:**
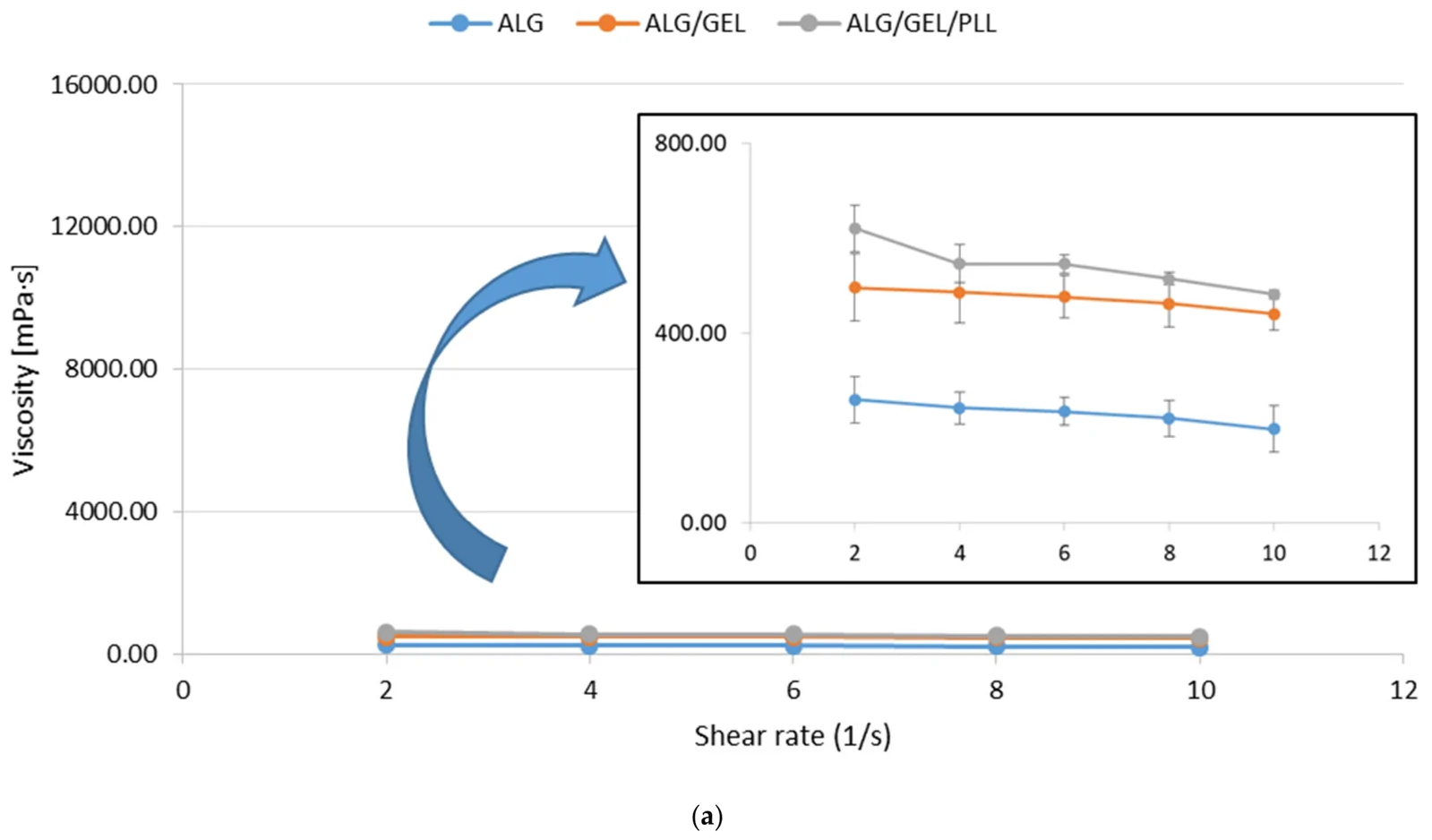

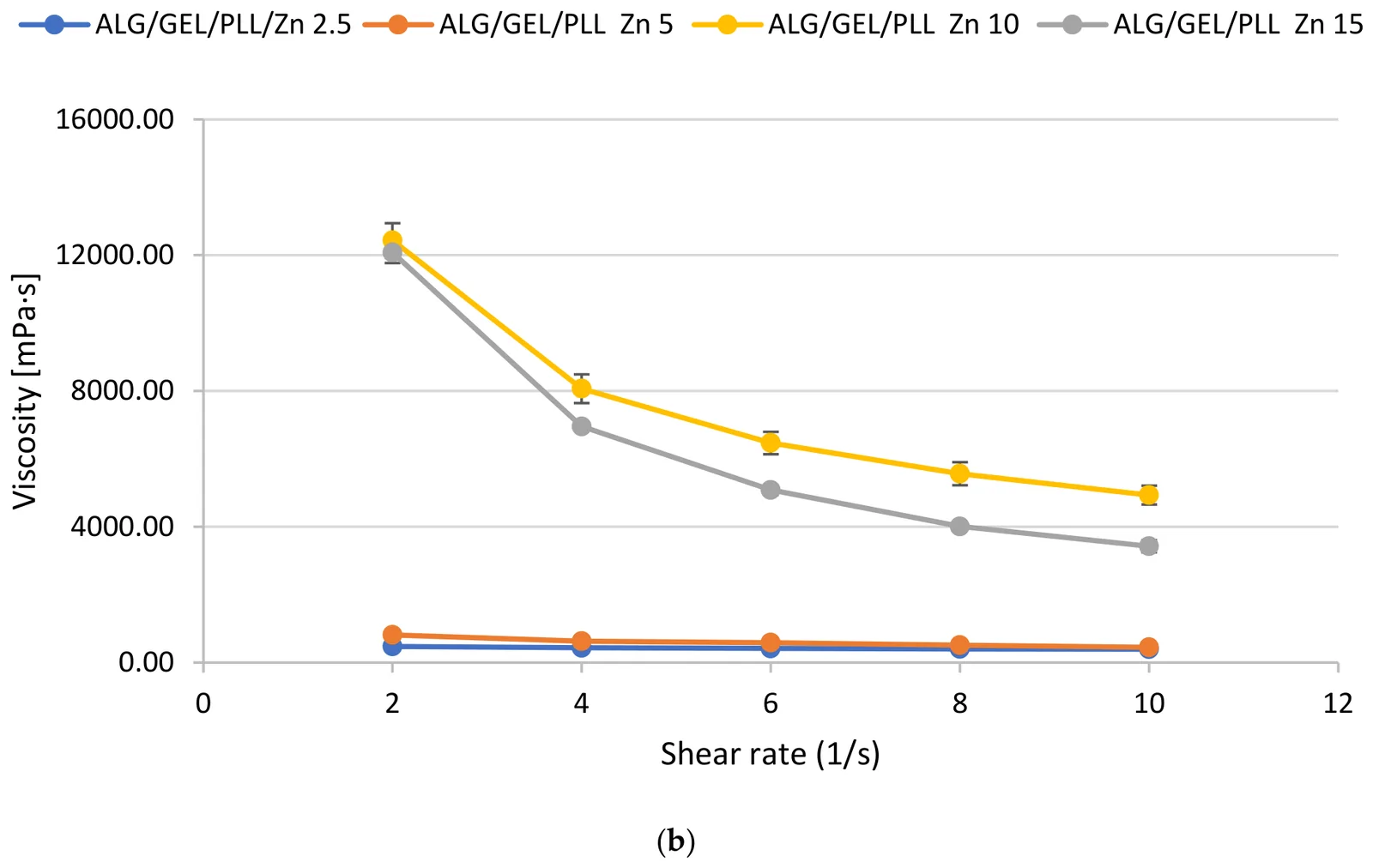
Viscosity plots versus shear rate of the (**a**) non cross-linked hydrogels, hydrogels cross-linked with GEL, dual cross-linked with GEL and PLL, and (**b**) triple cross-linked with Zn^2+^ ions with different volume of cross-linking agent measured at 22 ± 1 °C (mean ± SD, *n* = 3).

The assessment of rheological and textural characteristics is an essential tool for evaluating the structural organization of semisolid pharmaceutical formulations, including hydrogel systems. One of the fundamental techniques for characterizing rheological parameters is the determination of flow curves, which illustrate the relationship between dynamic viscosity and shear rate. The observed decrease in viscosity with increasing shear rate is typical of non-Newtonian fluids exhibiting pseudoplastic behavior, also referred to as shear thinning systems [24]. A thorough understanding of these parameters is crucial for optimizing technological processes such as homogenization, mixing, and dosing, as well as for selecting the appropriate packaging system and method of application. Experimental studies expressed that all the hydrogel formulations exhibited non-Newtonian behavior and belonged to the group of pseudoplastic systems, as confirmed by the rheological curves (Figure 4). At the same time, varying degrees of viscosity reduction were observed as a function of increasing shear rate. In the case of uncross-linked hydrogels, single GEL cross-linked hydrogels, and double cross-linked GEL/PLL hydrogels, the effect of viscosity reduction under increasing shear rate was negligible (Figure 4a). In the case of triple cross-linked hydrogels, only a slight decrease in viscosity was observed when 2.5 and 5 mL of a 3% cross-linking agent solution were used (Figure 4b). The decrease in viscosity under shear stress was particularly pronounced in samples cross-linked with 10 and 15 mL of a 3% Zn^2+^ ions, which may indicate the presence of pseudoplastic properties and the ordering of the system’s structure under the influence of shear forces (Figure 4b). ZnSO_4_ is a strong electrolyte, which dissociates into divalent Zn^2+^ and SO_4_^2−^ ions, provided to increase the ionic strength of the solution. This may lead to partial shielding (neutralization) of the electrostatic interactions between oppositely charged polymer chains. At the same time, Zn^2+^ ions act as a cross-linking agent, forming ionic-coordination bonds with the ALG carboxylate groups and contributing to the stabilization of the resulting polymer network. The final properties of the hydrogel result from the balance between the electrostatic interactions responsible for the formation of polyelectrolyte complexes between ALG, GEL, and PLL and the cross-linking of ALG by Zn^2+^. In the conducted studies, the application of 10 mL of 3% ZnSO_4_ solution ensured an adequate degree of ionic cross-linking while maintaining the proper balance between ALG cross-linking and the electrostatic interactions forming polyelectrolyte complexes. With a 2.5 and 5 mL solution of 3% ZnSO_4_ solution, the degree of ionic cross-linking was insufficient, resulting in too low a viscosity and insufficient stability of the gel structure. Conversely, increasing the amount of ZnSO_4_ led not only to more intense ALG cross-linking [25].

The analysis of textural properties is a major factor in assessing the performance qualities of hydrogels, particularly in terms of their storage, stability, and dosing from the packaging. This test involves recording the material’s response to mechanical force, with the results being directly determined by the internal structural organization of the system, resulting from the interactions between the formulation’s components [26]. The conducted analyses identified parameters such as hardness, consistency, and cohesion of the tested hydrogels. The firmness values and consistency were defined during the penetration of the sample by the measuring probe, while cohesion was calculated during the withdrawal phase, reflecting the structure’s ability to maintain its integrity. These features serve a practical purpose—lower hardness and consistency values facilitate dispensing the product from the packaging, ensure even distribution, and reduce difficulties associated with extracting the hydrogel from the container during long-term storage. In turn, higher cohesion indicates the system’s ability to rapidly reconstruct its structure after the removal of mechanical stress, which translates to maintaining physical stability and adequate functional quality of the preparation during application.

**Figure 5:**
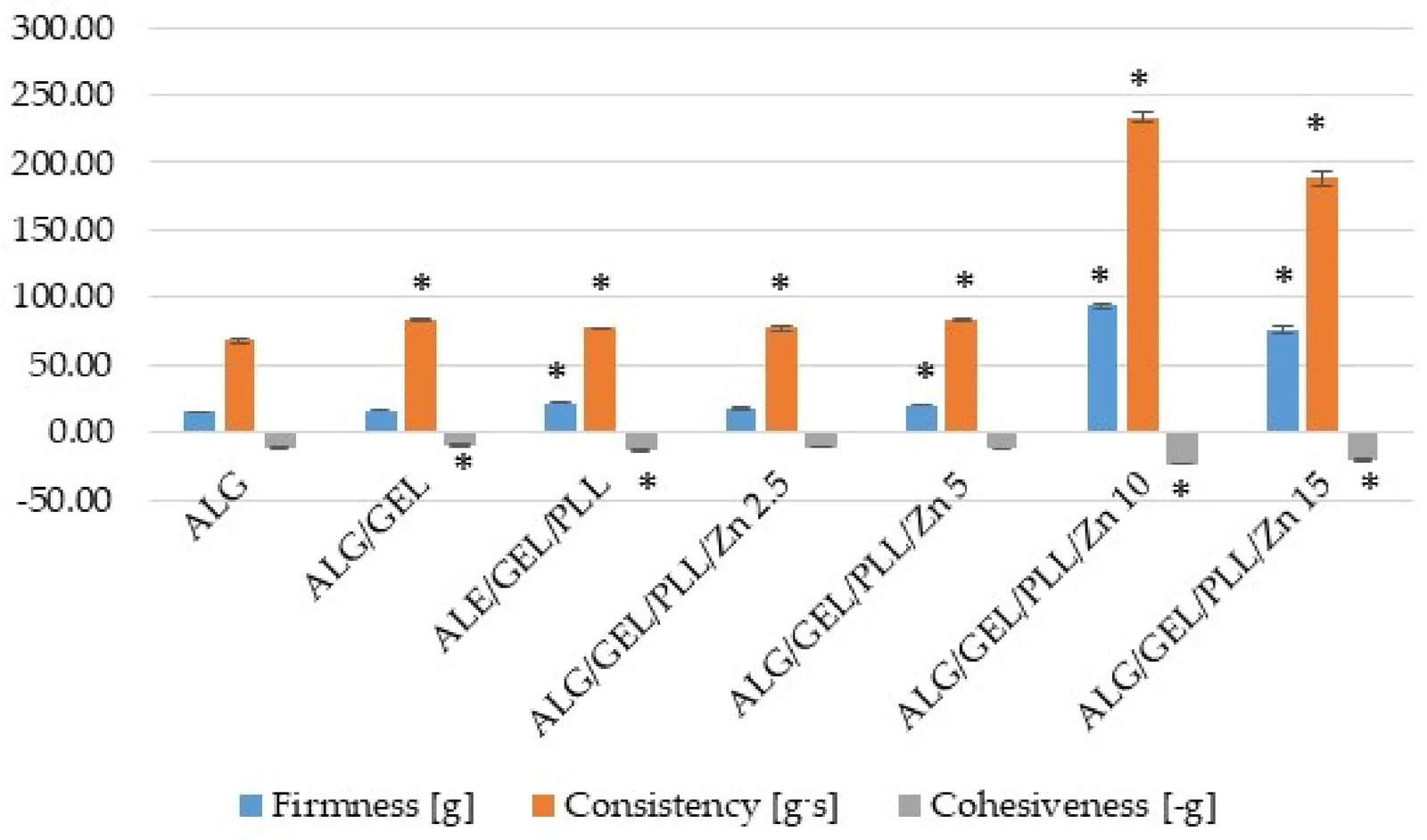
Textural properties of hydrogels basis: non cross-linked hydrogels, cross-linked with GEL, dual cross-linked with GEL and PLL, and triple cross-linked with Zn^2+^ ions with different volume of cross-linking agent (mean ± SD, *n* = 3, * significant statistical difference (*p* < 0.05) versus correspondent value of ALG solution).

Based on the conducted analysis, it was noted that the lowest parameters were observed in the non-cross-linked hydrogel, while the cross-linking process—whether double or triple—resulted in a linear increase in all parameters (Figure 5). It was demonstrated that the highest values for firmness, consistency, and cohesiveness were exhibited by the triple cross-linked formulation using 10 mL of a 3% Zn^2+^ ion solution. Using a higher-volume (15 mL) cross-linking agent resulted in a decrease in the analyzed parameters.

In summary, based on preliminary studies of the designed hydrogels to prepare mucoadhesive vaginal sponges via freeze-drying triple cross-linked ALG/GEL/PLL/Zn^2+^ hydrogels using 10 mL of a 3% Zn^2+^ solution were applied. These hydrogels’ formulations were characterized by a uniform, smooth texture and a viscosity that ensured homogeneous dispersion of the active ingredient and even dispensing into containers. At the same time, these formulations exhibited the most beneficial textural properties, including firmness, consistency, and cohesiveness.

In the next step, the all obtained sponges based on the non-cross-linked, ALG/GEL single cross-linked, ALG/GEL/PLL dual cross-linked, and ALG/GEL/PLL/Zn^2+^ triple cross-linked hydrogels were assessed for quality, during which the effect of the triple cross-linking process on their pharmaceutical, physicochemical, and biological properties was examined.

### 3.2. Sponges Quality Research

#### 3.2.1. Assessment of Sponge Structure and General Properties

**Figure 6:**
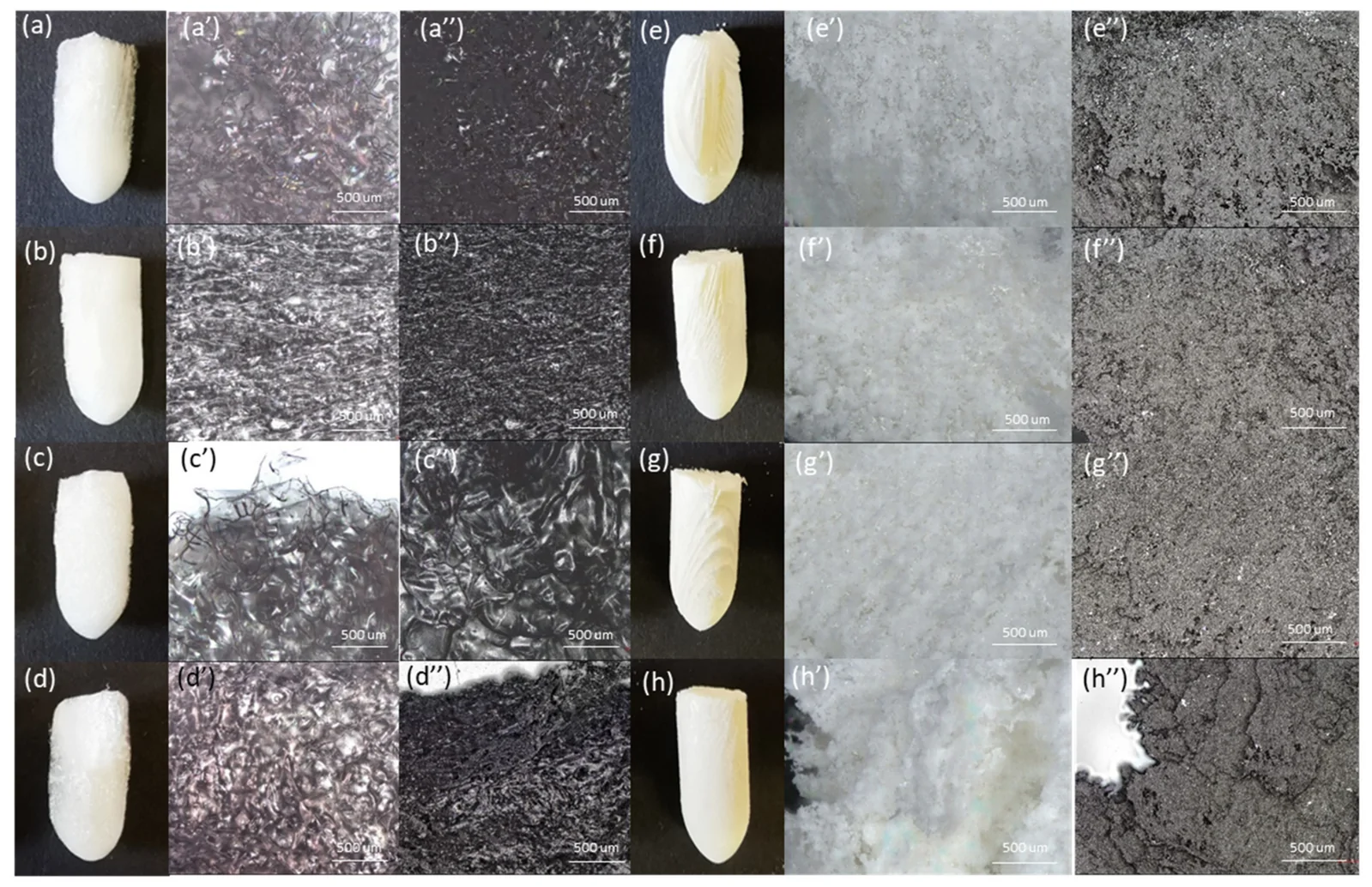
Example images of sponges: placebo non cross-linked and cross-linked (**a**) S0, (**b**) S1, (**c**) S2, and (**d**) S3 and with LUL (**e**) SL0, (**f**) SL1, (**g**) SL2, and (**h**) SL3 obtained with a camera, and representative images from confocal laser scanning microscope in optical (**a’**) S0, (**b’**) S1, (**c’**) S2, (**d’**) S3, (**e’**) SL0, (**f’**) SL1, (**g’**) SL2, and (**h’**) SL3 and laser-optical procedure (**a’’**) S0, (**b’’**) S1, (**c’’**) S2, (**d’’**) S3, (**e’’**) SL0, (**f’’**) SL1, (**g’’**) SL2, and (**h’’**) SL3 under magnification ×5.

**Figure 7:**
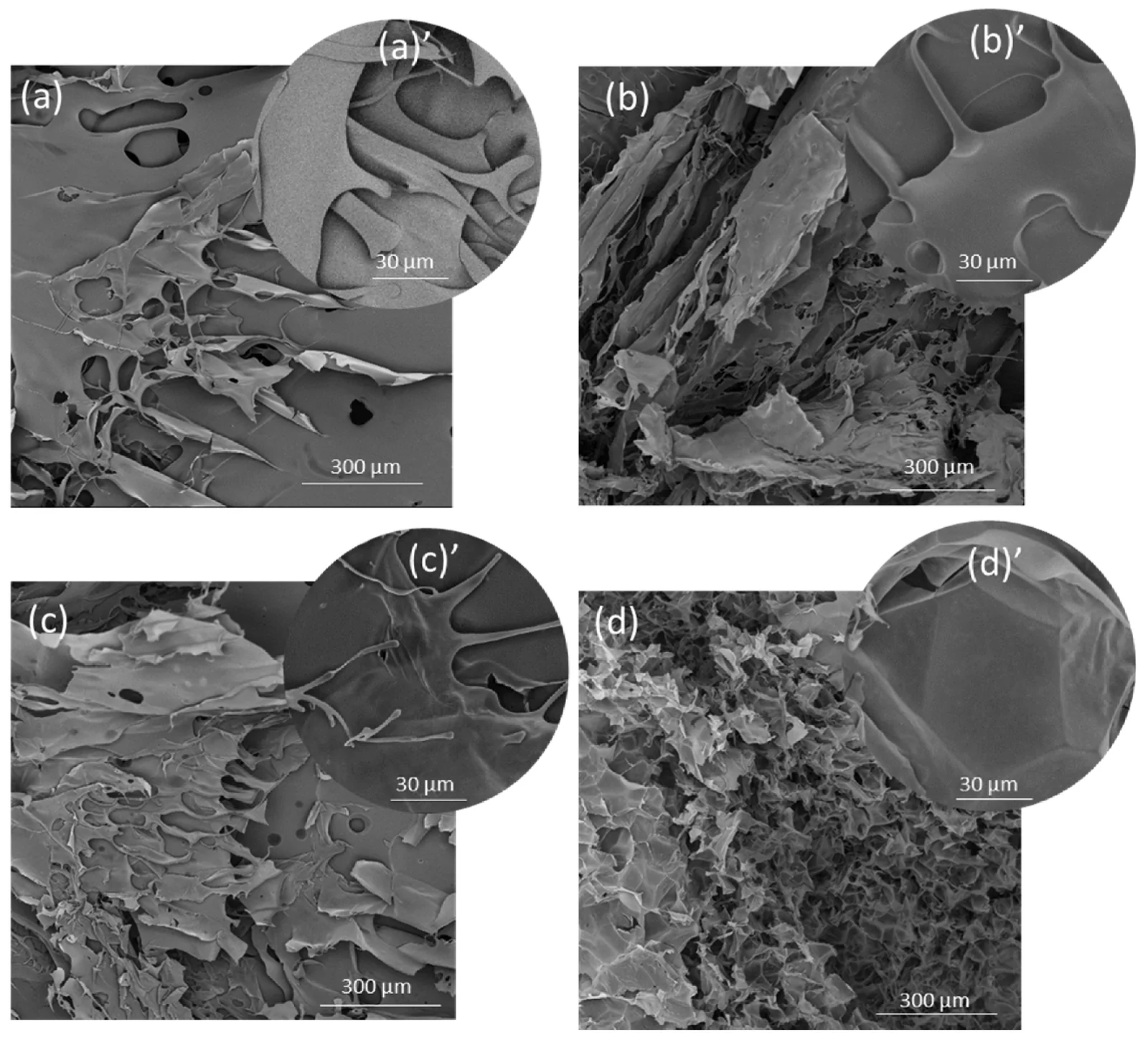
The representative SEM images of non-cross-linked and cross-linked sponges without LUL (**a**) S0, (**b**) S1, (**c**) S2, and (**d**) S3 under magnification ×200 and (**a’**) S0, (**b’**) S1, (**c’**) S2, and (**d’**) S3 under magnification ×2000.

**Figure 8:**
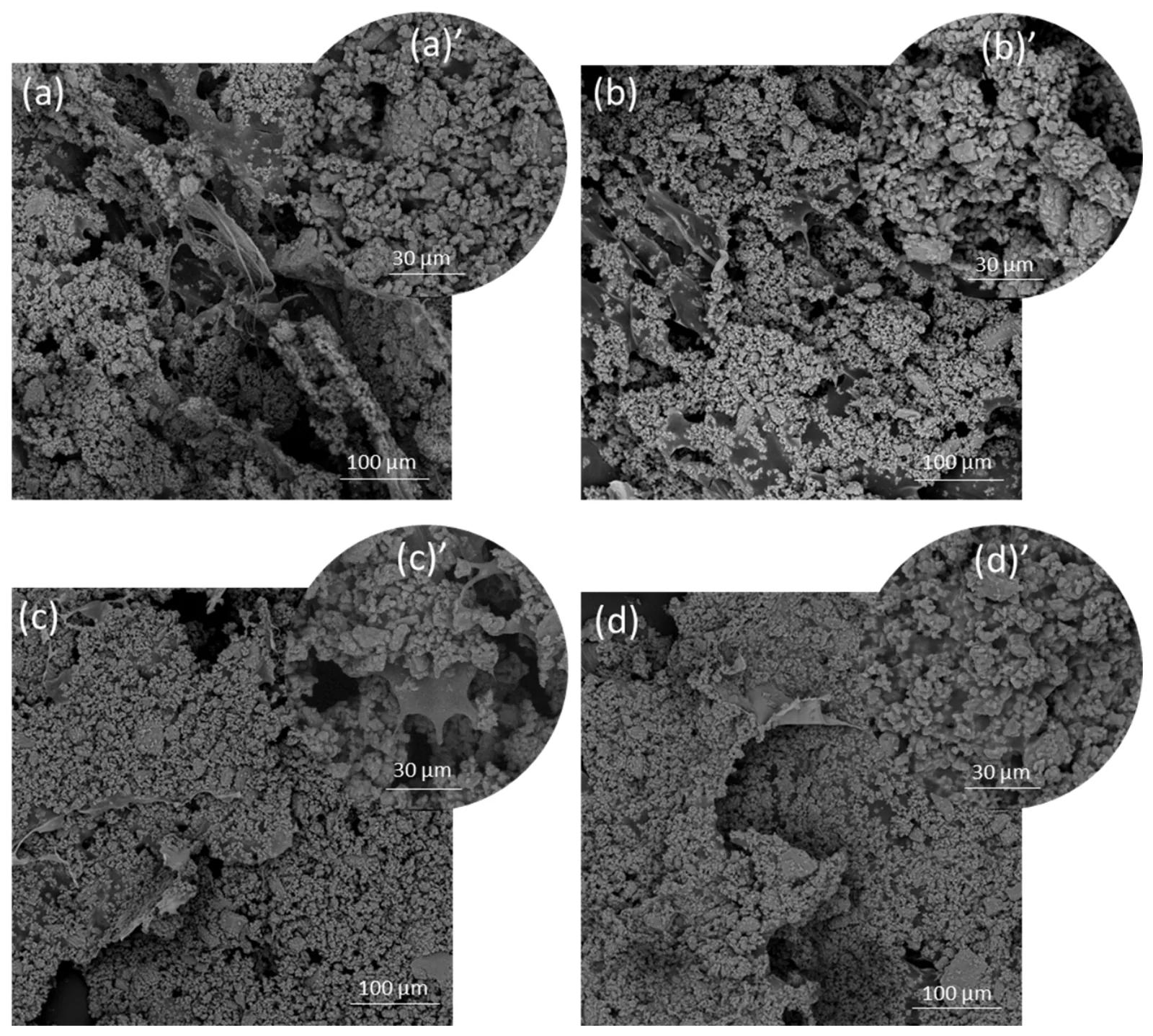
The representative SEM images of non-cross-linked and cross-linked sponges with LUL (**a**) SL0, (**b**) SL1, (**c**) SL2, and (**d**) SL3 under magnification ×500 and (**a’**) SL0, (**b’**) SL1, (**c’**) SL2, and (**d’**) SL3 under magnification ×2000.

Microscopic analysis with a scanning confocal laser microscope enabled evaluation of the surface morphology of the tested formulations, the uniformity of the structure, the presence of irregularities, surface defects, and topographical changes (Figure 6). The images obtained for the placebo formulations, for formulations S1 and S3, indicated the formation of a more compact and densely packed network as a result of cross-linking using GEL and Zn^2+^ ions. In the case of LUL-loaded formulations, the observed structure was distinctly dominated by the presence of drug particles. The structure of the received sponges, both the control and the formulations containing the active ingredient, was analyzed using scanning electron microscopy (SEM), and the resulting images are depicted in Figure 7 and Figure 8. The analyzed sponges exhibited a network structure; however, their microstructure underwent significant changes depending on the degree and type of cross-linking. It was found that the non-cross-linked formulation was characterized by a structure with irregular pores. In the case of ALG/GEL and ALG/GEL/PLL sponges, the formation of a jagged, dense microporous network with an irregular and partially flaking morphology was observed (Figure 7). This phenomenon is directly related to the formation of PEC between the components of the system. In the hydrogel environment, ALG carboxylate anions interact electrostatically with protonated amine groups—first with GEL during the initial cross-linking, and then with PLL—leading to the formation of PECs. As the cross-linking agents increase, complex formation intensifies, resulting in increased cross-linking density and gradual densification of the spatial structure, leading to further reorganization of the hydrogels’ microstructure. This facts were confirmed by He at al., who observed that as the carboxyl content (-COOH) increased in dual cross-linked sponge prepared by modifying collagen with dialdehyde oxidized cellulose, more PECs were produced and the cross-linked network structure became denser [27]. The synergistic action of electrostatic (with GEL and PLL) and ionic (with Zn^2+^) forces leads to the formation of a highly complex, three dimensional network with increased packing density. As a result of increased cross-linking, a significant reduction in the material’s porosity is observed, which stems from a decrease in interchain space and an increase in the structural order of the polymer network. High cross-linking density leads to the formation of a compact, highly condensed hydrogel structure in which the mobility of macromolecular chains is significantly limited. Similar findings were reported by Shen et al., who demonstrated that multi-step cross-linking leads to significant modifications in the microstructure of hydrogels. In his study, the combination of ALG dialdehyde (ADA) cross-linked using phenylboronic acid-grafted polyethyleneimine (PBA-PEI)-modified GEL (PPG), followed by the introduction of Ca^2+^ ions as an additional cross-linking agent, resulted in the formation of a triple cross-linked hydrogel network. The presence of imine, borate ester, and calcium carboxylate ionic interactions led to a significant increase in network density and a marked reduction in pore size [28]. Consequently, the resulting hydrogel exhibited the most compact and condensed structure among the analyzed systems, which confirms that the degree and mechanism of cross-linking play a key role in shaping the microstructural and functional properties of hydrogel materials. During the freeze-drying process, the dense hydrogel network formed in this way inhibits the growth and free sublimation of ice crystals within the matrix, resulting in the preservation of a finer and more homogeneous pore microstructure. Consequently, the resulting materials were characterized by reduced pore size and a more compact spatial architecture. In the case of LUL-loaded formulations, the presence of active ingredient crystals was identified (Figure 8).

The resulting formulations were characterized by homogeneity in terms of weight, length, and diameter, which demonstrates the reproducibility of the manufacturing process (Table 3). Furthermore, moisture content analysis revealed no moisture (0%), which may indicate the effectiveness of the drying process and the potentially favorable physicochemical stability of the resulting preparations.

The developed HPLC method for LUL determination was validated according to analytical validation requirements (ICH Q2(R2) Guideline Validation of Analytical Procedures) (Table S1). The method showed linearity over the concentration range of 5–100 µg/mL, with a regression equation of y = 0.513x − 0.1875 and a coefficient of determination (R^2^) of 0.9999. The accuracy of the method was 99.84%. The obtained precision results demonstrated good method repeatability (RSD = 0.006, CV = 0.60) and intra-day and inter-day precision, with CV values of 1.18 and 1.63, respectively. The calculated Limit of Detection (LOD) and Limit of Quantification (LOQ) values were 0.67 µg/mL and 2.03 µg/mL, confirming the sensitivity and applicability of the proposed method for LUL quantitative determination. Analysis of the active ingredient content expressed that its average concentration remained within the tolerance 90–110% limits specified by the USP [29], confirming the uniform distribution of LUL particles in the all tested sponges (Table 3).

#### 3.2.2. Sponges Mechanical Qualities

Solid vaginal drug formulations are expected to provide appropriately selected mechanical properties that ensure both comfort during application and therapeutic efficacy. Optimal hardness and mechanical strength are key, enabling the formulation to maintain its integrity during transport and application while avoiding excessive brittleness that leads to fragmentation. Adequate flexibility or plasticity is also essential, allowing the formulation to adapt to anatomical conditions and minimize user discomfort [30].

**Figure 9:**
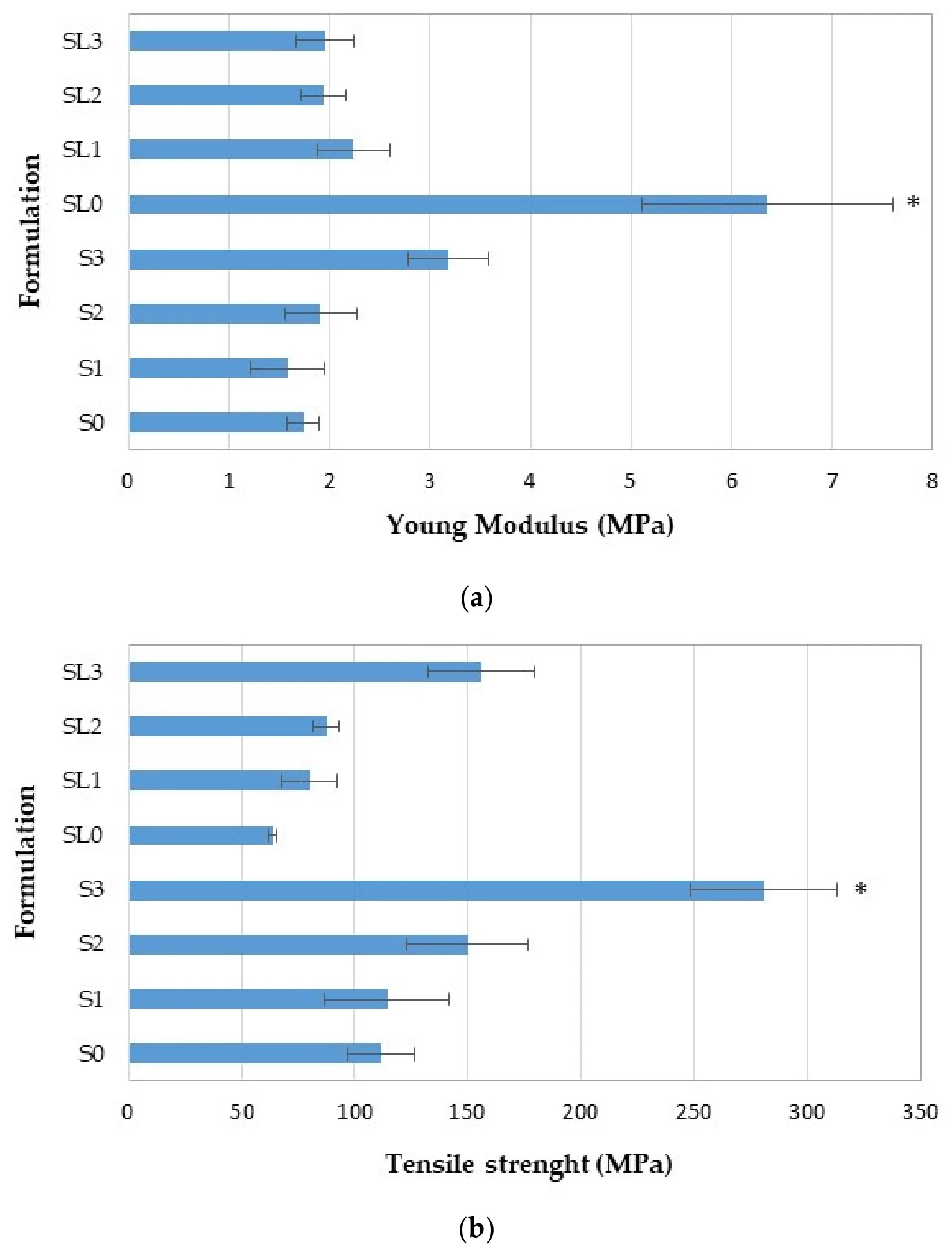

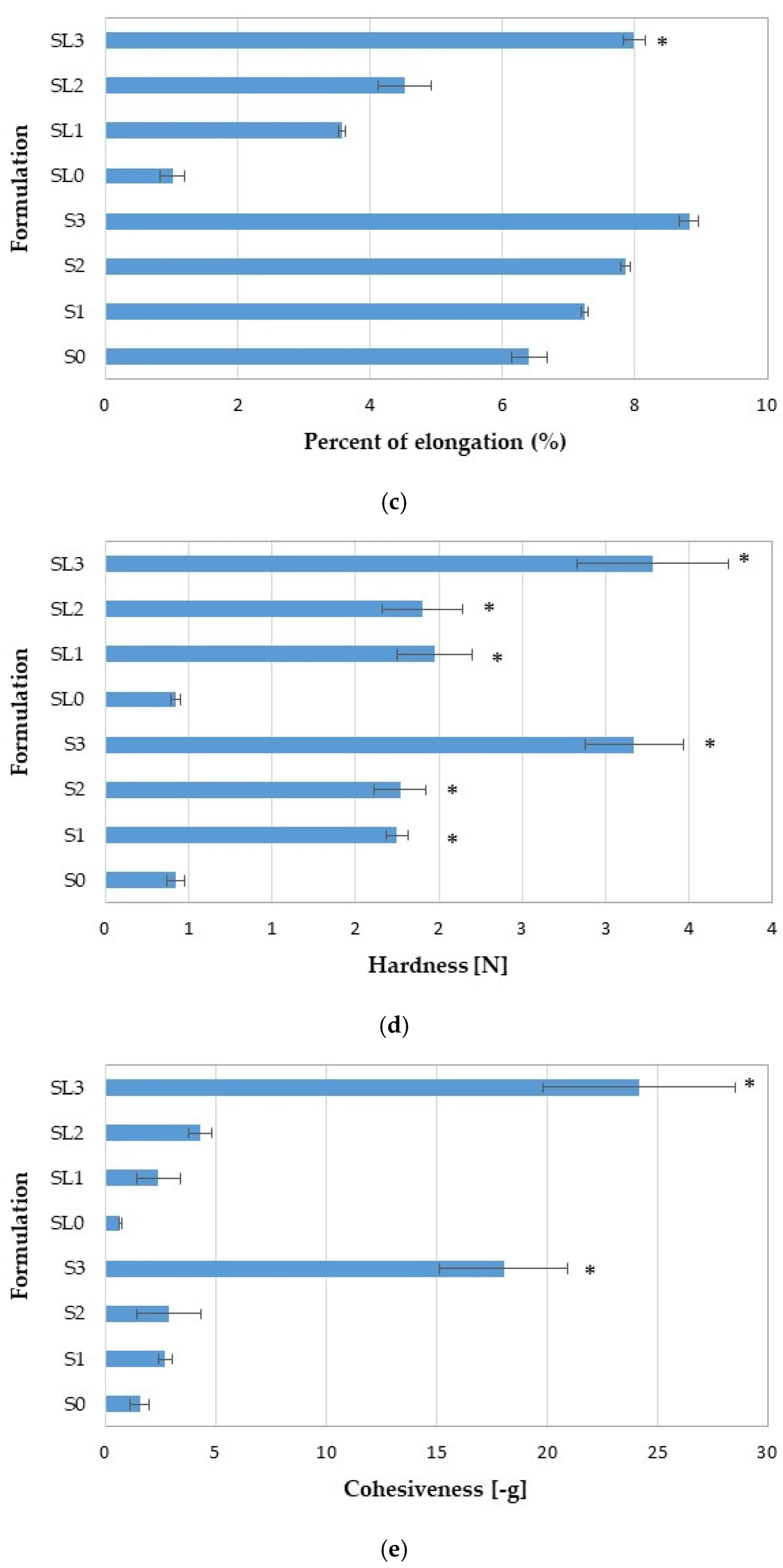
The mechanical properties of sponge placebo (S0–S3) and LUL-loaded formulations (SL0–SL3) expressed as (**a**) Young’s modulus, (**b**) tensile strength, (**c**) percent at elongation, (**d**) hardness, and (**e**) cohesiveness. * *p* < 0.05 versus S0.

The mechanical properties of the prepared sponges, both non-cross-linked and cross-linked, were evaluated based on parameters such as Young’s modulus, tensile strength, hardness, cohesion, and elongation at break, and the results of these parameters are presented in Figure 9. It was observed that the mechanical properties of the tested sponges showed a significant correlation with the degree of cross-linking and the presence of the active substance.

Young’s modulus, defined as the ratio of stress to strain, is a fundamental parameter describing a material’s stiffness—an increase in this value indicates greater resistance to deformation. The highest Young’s modulus was recorded for SL0, where it was 6.35 ± 1.25 MPa (Figure 9a). For the placebo formulations, the lowest value was noted for S0 (1.74 ± 0.17 MPa) and the highest for S3 (3.19 ± 0.40 MPa). The addition of LUL increased Young’s modulus as cross-linking progressed, whereas in the case of the S3 and SL3 formulations, it caused a decrease.

Tensile strength is a measure of the maximum stress a material is able to withstand when stretched before breaking. This parameter reflects the material’s resistance to tensile forces and is of significant importance in assessing its mechanical strength. The results suggest that as the degree of cross-linking increases, tensile strength gradually increases, indicating a reinforcement of the polymer structure (Figure 9b). As expected, the lowest tensile strength values were recorded for the uncross-linked samples S0 (111.63 ± 15.16 MPa) and SL0 (63.40 ± 1.81 MPa). The highest data were noted for samples with the highest degree of cross-linking, i.e., S3 (280.85 ± 32.19 MPa) and SL3 (156.20 ± 23.69 MPa). It was found that the addition of the therapeutic agent influenced the reduction in the measured parameter.

The hardness of the tested materials, defined as resistance to local plastic deformation, also increased with the degree of cross-linking. The lowest values were observed for non-cross-linked samples, while the highest were for systems with the highest degree of cross-linking, with the presence of the drug causing a slight increase in this parameter (Figure 9c). The cross-linking process increases hardness, with the lowest values for S0 (0.42 ± 0.05 N) and SL0 (0.43 ± 0.03 N), and the highest for S3 (3.17 ± 0.29 N) and SL3 (3.28 ± 0.45 N) formulations. Incorporation of the active substance causes a slight increase in the hardness of the analyzed sponges.

Cohesion, interpreted as a measure of the internal cohesive forces between polymer chains, expressed a similar trend—it increased linearly as the degree of network connectivity increased—from 1.57 ± 0.41 -g in formulation S0 and 0.69 ± 0.06 -g in SL0 to 18.06 ± 2.91 -g in formulation S3 and 24.20 ± 4.35 -g in SL3 (Figure 9d). This is due to chemical bonds (ionic, covalent, and metallic) and van der Waals interactions. High cohesion means that the material is difficult to tear or crush and indicates greater structural integrity of the material and its resistance to tearing.

Elongation percentage is a measure of a material’s ductility, defined as the ratio of the increase in the measured length of a specimen after fracture to its initial length, expressed as a percentage. It is a key parameter in material strength testing during static tensile testing. A higher percentage value indicates that the material is more ductile (capable of plastic deformation before fracture), while a low value suggests a brittle material (Figure 9e). In turn, the elongation at break—a measure of the material’s ductility—increased with the degree of cross-linking (6.40 ± 0.27% in S0 and 1.02 ± 0.19% in SL0), reaching the highest values for the most cross-linked samples (8.82 ± 0.15% in S3 7.99 ± 0.16% in SL3). At the same time, the addition of the active substance caused a slight decrease in this value, which may indicate partial stiffening of the structure and a reduction in the mobility of the polymer chains.

In summary, the degree of cross-linking and the presence of the active substance significantly determine the mechanical properties of the tested formulations, influencing the balance between their stiffness, strength, and elasticity, which is of key importance for their potential application in vaginal drug delivery systems. Cross-linking agents perform a crucial role in modifying the mechanical properties of ALG-based drug delivery systems by modulating the density and nature of the polymer network. In the case of ionic cross-linking, polyvalent cations, such as Zn^2+^ ions, form ordered “egg-box” structures with ALG G-blocks, leading to an increase in the elastic modulus and stiffness of the gel. This effect depends on the type and concentration of the ions and the strength of their interactions with the polymer chains. In turn, the formation of polyelectrolyte complexes with opposite charged polymers such as PLL and GEL is based on electrostatic interactions between oppositely charged functional groups, resulting in a more compact and cross-linked structure that restricts chain mobility and increases mechanical strength and resistance to deformation [31]. The combination of triple cross-linking in the developed sponges—consisting of double cross-linking based on the formation of polyelectrolyte complexes and ionic cross-linking using Zn^2+^ ions—leads to an increase in network density and the number of intermolecular interaction sites. This results in increased stiffness, strength, and mechanical stability of alginate systems, while simultaneously affecting their elasticity [32]. Saravanou et al., who demonstrated the synergistic effect of three cross-linking interactions in a triple cross-linked hydrogel based on a synthetic, doubly grafted NaALG-g1-P(NIPAM86-co-NtBAM14)-g2-BA (DG) network, utilized gelation via three different cross-linking mechanisms: hydrophobic association of thermoresponsive side chains, dynamic/reversible formation of boronic esters at alkaline pH, and ionic cross-linking using Ca^2+^ ions, which contributed to a mechanically stronger polymer network [33].

#### 3.2.3. Sponges Mucoadhesive Properties

Mucosal adhesion is a phenomenon based on the adhesion of polymers—both synthetic and natural—to the surface of the mucous membrane. In the context of intravaginal drug delivery, it plays a significant role in determining therapeutic efficacy. This mechanism provides prolonged contact between the formulation and the target tissue and limits the action of the vagina’s physiological self-cleansing process, which promotes the controlled and prolonged release of active substances [4].

**Figure 10:**
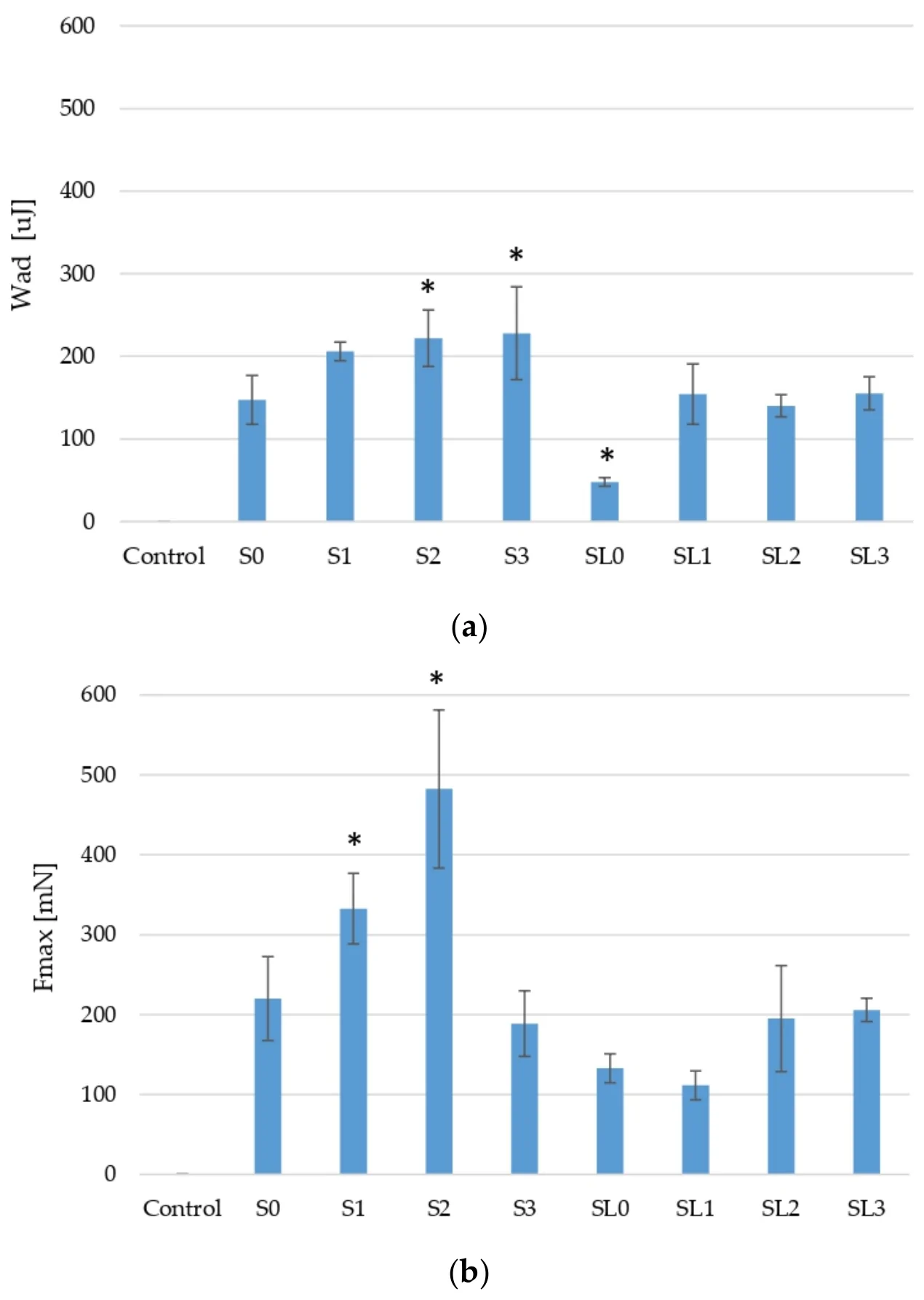
Mucoadhesiveness of sponge placebo (S0–S3) and LUL-loaded formulations (SL0–SL3) expressed as (**a**) W_ad_ and (**b**) F_max_ and cellulose disc (control) (mean ± SD, *n* = 6, significant statistical difference (*p* < 0.05) * compared to correspondent value of S0).

An analysis of mucoadhesive properties, expressed as adhesion forces and work, expressed that all formulations exhibited adhesion to the porcine vaginal mucosa (Figure 10). In placebo formulations, an increase in work of adhesion was observed as the degree of cross-linking increased, reaching values ranging from 147.27 ± 29.43 µJ (in formulation S0) to 227.93 ± 56.11 µJ (in formulation S3) (Figure 10a). Adhesion force data also expressed an upward trend—it rises linearly in successive formulations from 220.14 ± 52.63 N to in formulation S2, and followed by a significant decline to a level of 188.80 ± 40.93 N in S3 formulation (Figure 10b). The effect of the active ingredient on the adhesive properties of the dosage form depends on a number of physicochemical factors, such as solubility, electrical charge, polarity, particle size, and compatibility with the other components of the formulation. Formulations containing LUL exhibited varying effects on adhesive properties depending on the degree of cross-linking. In sponges containing GEL, a slight decrease in adhesive strength was observed alongside an increase in adhesive work, which may indicate an enhanced ability of the formulation to maintain contact with a biological surface. In contrast, the addition of PLL led to an increase in adhesion force, while the work of adhesion decreased slightly, suggesting the dominance of electrostatic interactions in the initial phase of contact. The highest values of the analyzed parameters were noted for preparation SL3, which achieved F_max_ of 205.86 ± 14.15 N and W_ad_ a 155.19 ± 19.95 µJ. This indicated the presence of a synergistic effect and enhanced mucoadhesive properties.

ALG carboxyl groups, an anionic polysaccharide, are capable of forming hydrogen bonds with the positively charged hydroxyl groups of mucus glycoproteins, which accounts for the phenomenon of mucoadhesion. Additionally, the polymer’s ability to swell and form hydrogels increases the contact surface with the tissue, enhancing the mucoadhesive effect. GEL, a naturally occurring protein, exhibits an amphoteric nature [19]. Compared to strong covalent interactions, it causes weak electrostatic interactions with mucosal surfaces [34]. However, its gelling properties and ability to form elastic three-dimensional networks promote tight adhesion to the epithelium, and thus it is used as an adhesion promoter [35]. PLL, as a cationic peptide polymer, exhibits high mucoadhesive properties, primarily due to the presence of numerous positively charged amino groups. These interact electrostatically with the negatively charged sialic acid and sulphate residues present in the structure of mucins. Additionally, hydrogen bonding and ionic interactions might form, which enhances adhesion to the mucosal surface. As a result, PLL might increase formulation retention at the application site and improve the bioavailability of active ingredients. Zn^2+^ cations, due to their positive charge, can additionally play a significant role in intensifying the mucoadhesion phenomenon. Despite the partial restriction of free carboxyl groups in Zn^2+^ cross-linked ALG, the gel structure still allows for the formation of hydrogen bonds and electrostatic interactions with mucins. This was confirmed by Dong et al., who proved that GEL and Zn^2+^ ions present in triple cross-linked p(am-co-aa) hydrogels (acrylamide-acrylic acid copolymer/GEL/Zn^2+^) provide perfect adhesion regardless of physical activity [36]. Similarly, Ponedel’kina et al. demonstrated the positive effect of Zn^2+^ ions on the mucoadhesive properties of zinc hyaluronate, which significantly enhanced the contact between the substance and biological surfaces compared to sodium hyaluronate [37]. In addition, Zhang et al. reported a synergistic effect of triple cross-linking. They demonstrated that a triple cross-linked hydrogel with a dual alginate/dextran/dendrimer network exhibits excellent adhesive properties both in vitro and in vivo on human corneal tissues [6].

**Figure 11:**
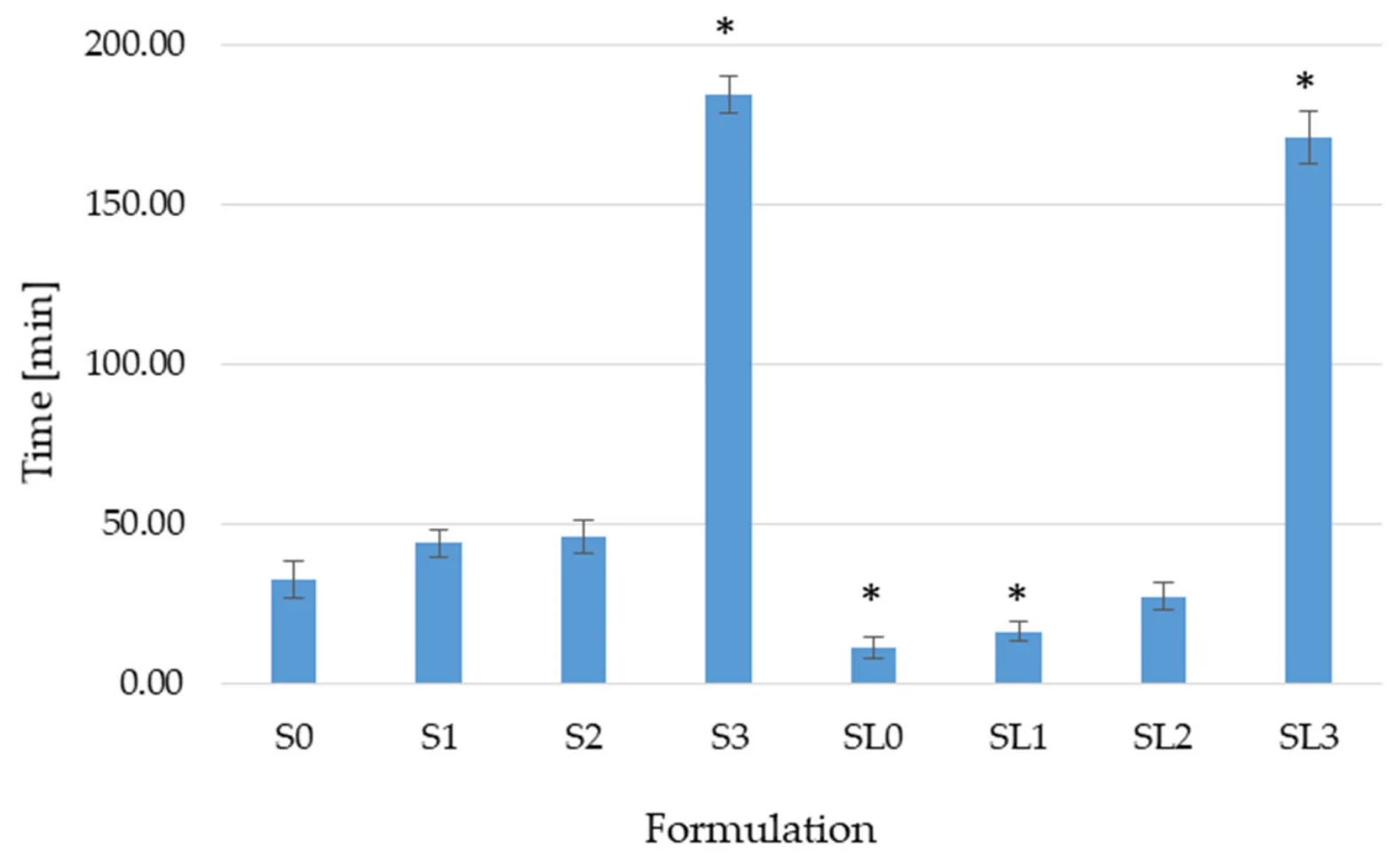
Ex vivo residence time of sponge placebo (S0–S3) and LUL-loaded formulations (SL0–SL3) (mean ± SD, *n* = 3, * significant statistical difference (*p* < 0.05) compared to correspondent value of S0).

Ex vivo residence time studies on the mucous membrane are a crucial component of the evaluation of formulations intended for vaginal administration, allowing for the determination of their ability to remain at the site of administration. Extended retention time enables a drug’s prolonged release, increases its local concentration, and improves therapeutic efficacy [3]. In the conducted studies, it was observed that the cross-linking process led to an increase in retention time on the mucosal membrane, indicating a beneficial effect of polymer structure modification on the mucoadhesive properties of the formulation (Figure 11), although the addition of the active substance resulted in a reduction in ex vivo retention time. The formulation with the triple cross-linking degree was characterized by a statistically significantly prolonged ex vivo retention time, both for placebo and active substance-loaded formulations. The results suggest that a high degree of cross-linking provided beneficial adhesive properties even after the incorporation of the active ingredient.

#### 3.2.4. Sponges Swelling and LUL In Vitro Release

**Figure 12:**
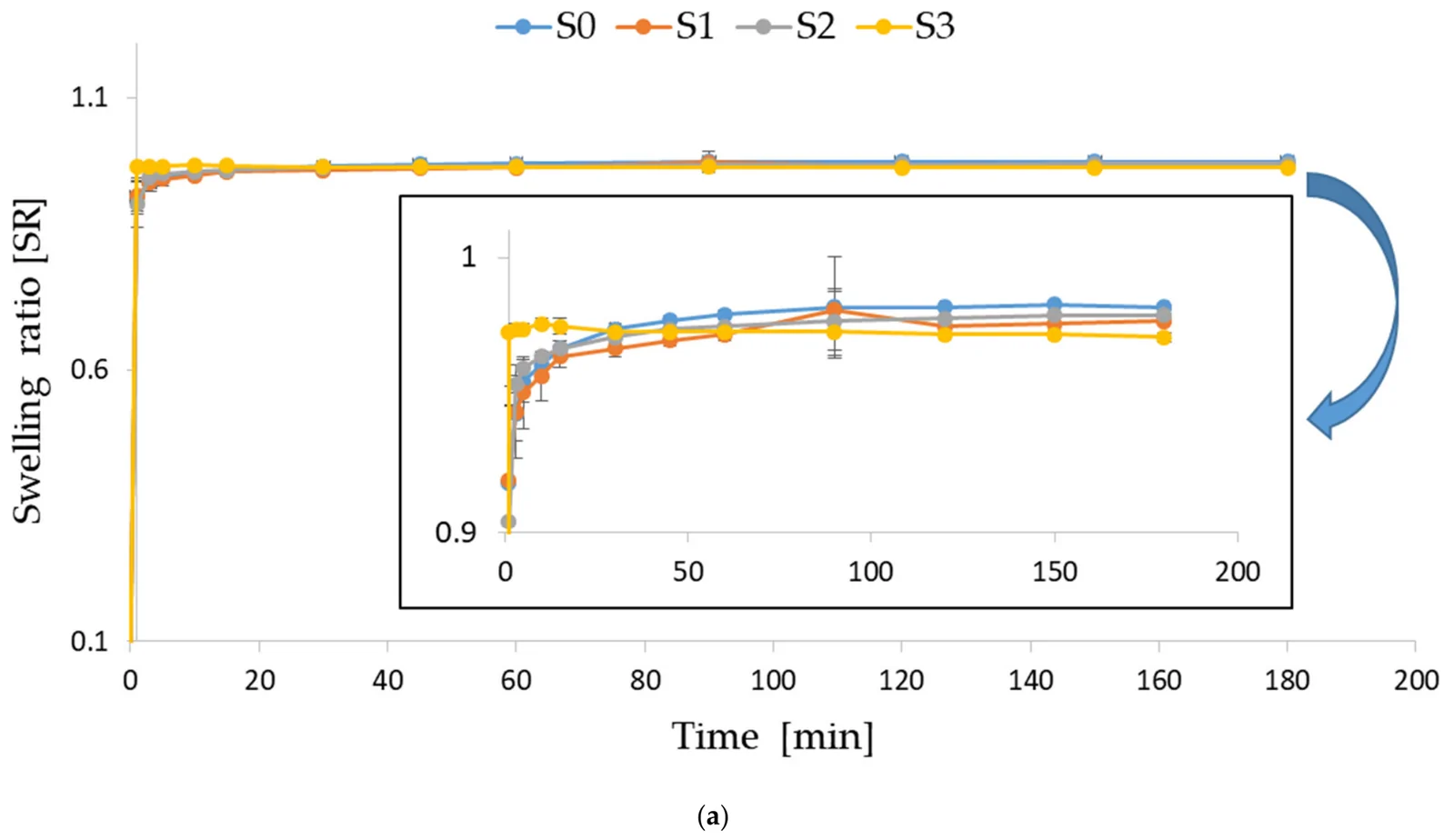

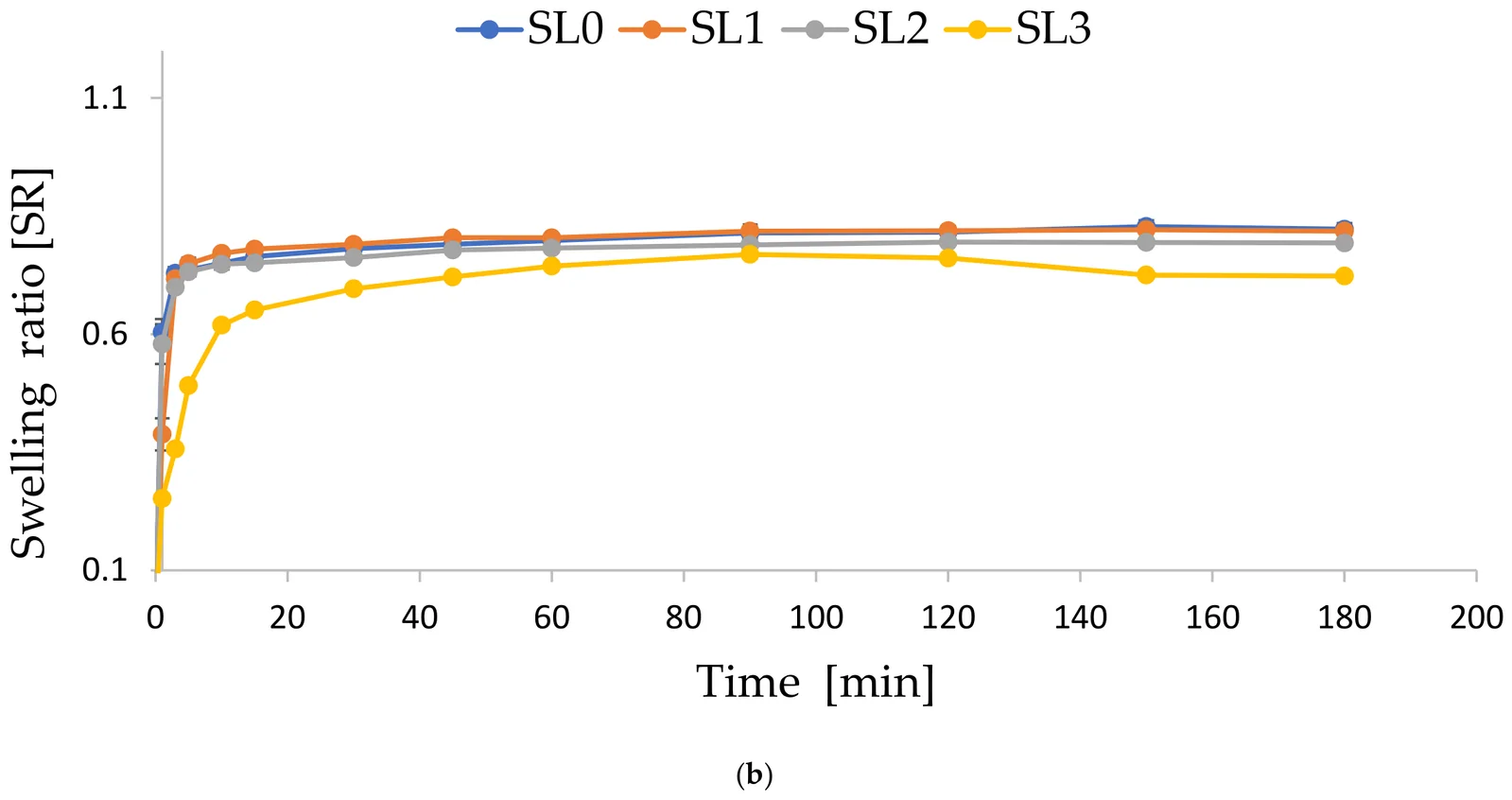
Swelling properties of (**a**) sponge placebo (S0–S3) and (**b**) LUL-loaded formulations (SL0–SL3) (mean ± SD, *n* = 3).

As a hydrophilic polysaccharide containing numerous carboxyl groups, ALG possess the ability to bind large amounts of water and form a hydrated, three-dimensional hydrogel network. The degree of swelling depends on factors such as the pH of the environment, ionic strength, and the presence of cross-linking ions, which modulate the density and stiffness of the polymer structure. ALG swelling is a decisive factor in terms of its mucoadhesive properties and release profile [38]. Hydration of the polymer increases its flexibility and provides better adaptation to the uneven surface of the mucosal membrane, increasing the contact area and facilitating the formation of hydrogen bonds and electrostatic interactions with mucins. However, excessive swelling may lead to a weakening of the gel’s structural integrity and a reduction in adhesion strength due to the loosening of the polymer network. The swelling of ALG also has a direct impact on drug release kinetics. An increase in the degree of hydration promotes the diffusion of drug molecules through the hydrogel matrix, leading to their gradual and controlled release. Simultaneously, a more compact and highly cross-linked structure limits the rate of water penetration into the formulation and the diffusion of the active ingredient, thereby prolonging the release time of the active ingredient. As a result, proper control of the degree of swelling enables optimization of both mucoadhesive properties and the drug release profile [39]. The analysis of the swelling properties of all prepared sponges was conducted in a simulated vaginal buffer (SVF, pH 4.2), and the results are presented in Figure 12. It was shown that in the placebo formulations, very similar values of the analyzed parameters were found (Figure 12a). Formulation S3 exhibited the most rapid initial swelling rate (0.973 ± 0.002 after 1 min), reaching a high level point within the first 15 min (0.975 ± 0.003), then followed by a stabilization phase. In contrast, formulation S1 was distinguished by the slowest, 0.904 ± 0.042, gradual, and more uniform absorption of the medium, ultimately reaching the highest values for this parameter at the 90 min point (0.977 ± 0.011). Slight differences in the behavior of the formulations during analysis suggest that the observed effect does not result directly from the degree of cross-linking of the system, but may be determined by the properties of the acidic medium used. In low-pH environments, the carboxyl groups of ALG undergo protonation, which reduces the degree of polymer ionization and limits its swelling. ALG is an anionic polysaccharide, the physicochemical properties of which are highly dependent on the pH of the environment. At lower pH levels, a more compact gel structure might form as a result of increased hydrogen bonding between polymer chains. These phenomena results in reduced permeability of the matrix and slowed diffusion [40]. Consequently, in an acidic environment, ALG tends to exhibit limited swelling, which might translate into a more controlled, prolonged release of incorporated compounds. In the case of drug-loaded sponges, differences in swelling rates are more noticeable within the formulation (Figure 12b). Swelling degree evaluation revealed a similar swelling profile for formulations SL0 and SL1, indicating similar kinetics of medium absorption. Sample SL2 was characterized by a more extended influx of medium into the polymer structure. However, formulation SL3 exhibited a progressive increase in the degree of swelling from 0.252 ± 0.002 in the first min up to approximately 90 min (0.769 ± 0.008), followed by a stabilization phase with values of 0.723 ± 0.002 after 180 min. The most gradual rate of swelling in this case is associated with a higher degree of cross-linking, which limits the dynamics of medium penetration into the interior of a more compact structure. Similar results were obtained by Liu et al., who analyzed the swelling rate of a wound dressing based on a combination of chitosan methacrylate (CTSMA) and chitosan hydrogen sulfide (CTSSH) cross-linked simultaneously via a free-radical polymerization reaction and a thiol reaction. They showed that the double cross-linked formulations were characterized by lower swelling capacity due to the strong cross-linking, which created a dense structure that hindered the inflow of the medium into the dressing [41]. Generally, the reduced water binding capacity results from the increased involvement of hydroxyl (–OH) and carboxyl (–COOH) groups in the formation of polymer networks with cross-linking agents. Consequently, a limited number of these groups remain available to form hydrogen bonds with water molecules, leading to a reduction in the matrix’s overall water sorption capacity, whereas the reduced swelling of the formulation compared to the placebo formulation might be attributed to the limited diffusion of solvent molecules through the densely cross-linked polymer network. Additionally, the poor solubility of the active substance in the medium limits its interaction with the solvent, which results in a lower water absorption capacity. As a result, both of these factors synergistically contribute to the reduction in swelling of the tested formulations [42,43].

The effect of polymer swelling on drug release is critical, and swelling assessment enables the prediction release profile of the API from the analyzed formulation. As swelling capacity increases, the polymer undergoes hydration and structural loosening, which increases its porosity and chain mobility, thereby facilitating the diffusion of drug molecules into the external environment. As a result, a higher degree of swelling typically accelerates the release of the active substance. Conversely, limited swelling leads to the maintenance of a compact matrix structure, which reduces the rate of solvent penetration and slows down the release. Swelling may also initiate polymer erosion or relaxation mechanisms, which further modulate the release profile [44].

**Figure 13:**
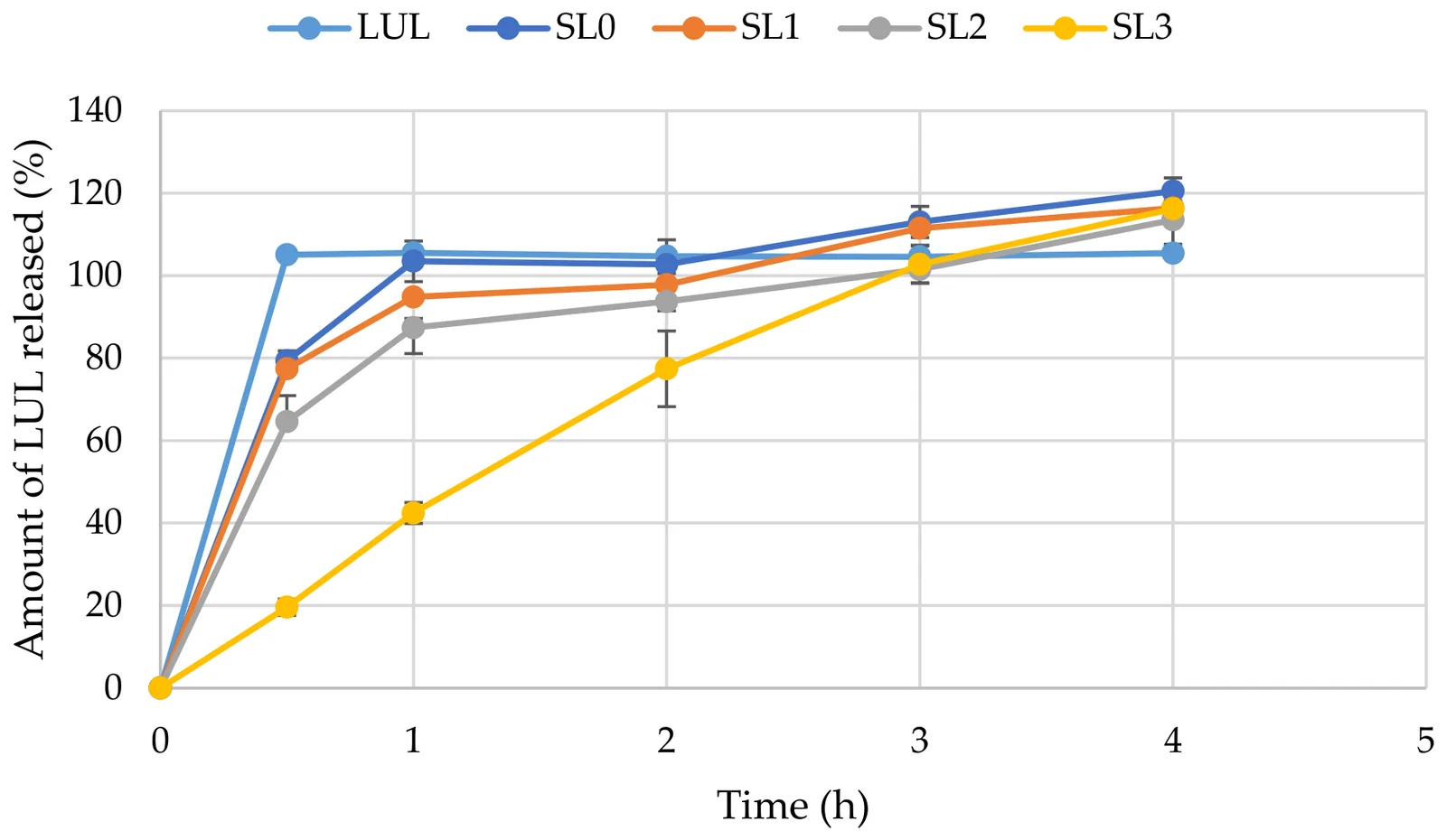
LUL release from sponge formulations SL0–SL3 (mean ± SD, *n* = 3).

The LUL release profiles of the received sponges in SVF solution at pH 4.2 are illustrated in Figure 13. In vitro studies revealed an initial “burst release” effect after 30 min, with values ranging from 19.57 ± 1.98% for formulation SL3 to 77.41 ± 0.54% for formulation SL1, followed by a gradual, controlled release that remained constant throughout the experiment. The initial release phase might be attributed to the dissolution of the drug fraction adsorbed onto the sponges’ surface. However, the prolonged LUL release phase resulted from the presence of active substance entrapped within the cross-linked polymer structure. The unmodified SL0 formula demonstrated the release of over 100% of the substance just one hour after the initial analysis. However, in the case of cross-linked formulations, extended LUL release was noted. It was observed at 94.82 ± 0.36% in formulation SL1, 87.36 ± 6.29% in SL2, and 42.44 ± 2.54% in SL3 drug release after 1 h. As the degree of matrix cross-linking increased, the release profile prolonged. The most extended profile was achieved for formulation SL3, which is consistent with the results of the swelling analysis. The degree of polymer cross-linking is one of the key factors determining the kinetics of active ingredient release from matrix systems. As the cross-linking density increases, the mobility of the polymer chains is restricted, which leads to a reduction in the system’s swelling capacity. Consequently, the diffusion of drug molecules through the matrix is significantly delayed. This indicates that double and triple cross-linked structures were characterized by increased mechanical stability and lower susceptibility to erosion, which further limited the release rate. In addition, the compact, cross-linked structure reduced the formulation’s permeability into the aquatic environment, increased the diffusion distance, and ensured a more controlled release profile. As a result, increasing the degree of cross-linking leads to a prolonged and more uniform release of the active ingredient.

The LUL kinetics release from the hydrogel formulations were analyzed utilizing a variety of mathematical models (Table 4). Linear regression analysis indicated that, for formulations SL0–SL2, the release process followed zero-order kinetics, whereas for formulation SL3, first-order kinetics provided the most accurate description. High values of the R^2^ coefficient in the Higuchi model suggest that diffusion was the dominant release mechanism. Additionally, the release mechanism was evaluated with the Korsmeyer–Peppas model. The calculated values of the exponent *n* ranged from 0.12 to 0.59. For formulations SL0–SL2, the *n* values were in the range of 0.12 to 0.17 (Table 4), and these values indicate Fickian diffusion (Case I transport) controlled primarily by the concentration gradient. In contrast, formulation SL3 reached a n value equal to 0.59, which falls within the range 0.45 < n < 0.89; a non-Fickian (anomalous) mechanism was observed, suggesting the combined contribution of diffusion and matrix swelling in the active ingredient release process. Increasing the degree of cross-linking in the polymer matrix leads to a change in the transport mechanism of the active substance—from a mechanism dominated by Fickian diffusion to anomalous transport, in which both diffusion processes and the relaxation and reorganization of the polymer network play a significant role [26]. This fact was observed by Jabeen et al., who prepared a bead in hydrogel (BIH) system based on a hydrogel matrix composed of Acacia gum, sodium alginate, and poly(vinylpyrrolidone) (PVP) modified by multiscale cross-linking with (3-glycidyloxypropyl)trimethoxysilane (GPTMS) and functionalized with *Alkanna tinctoria* (Ratanjot, RJ) extract. They found that the denser network structure in cross-linked formulations shifts the release mechanism from a diffusive behavior toward an anomalous (non-Fickian) one [27].

#### 3.2.5. Thermal Analysis Results

**Figure 14:**
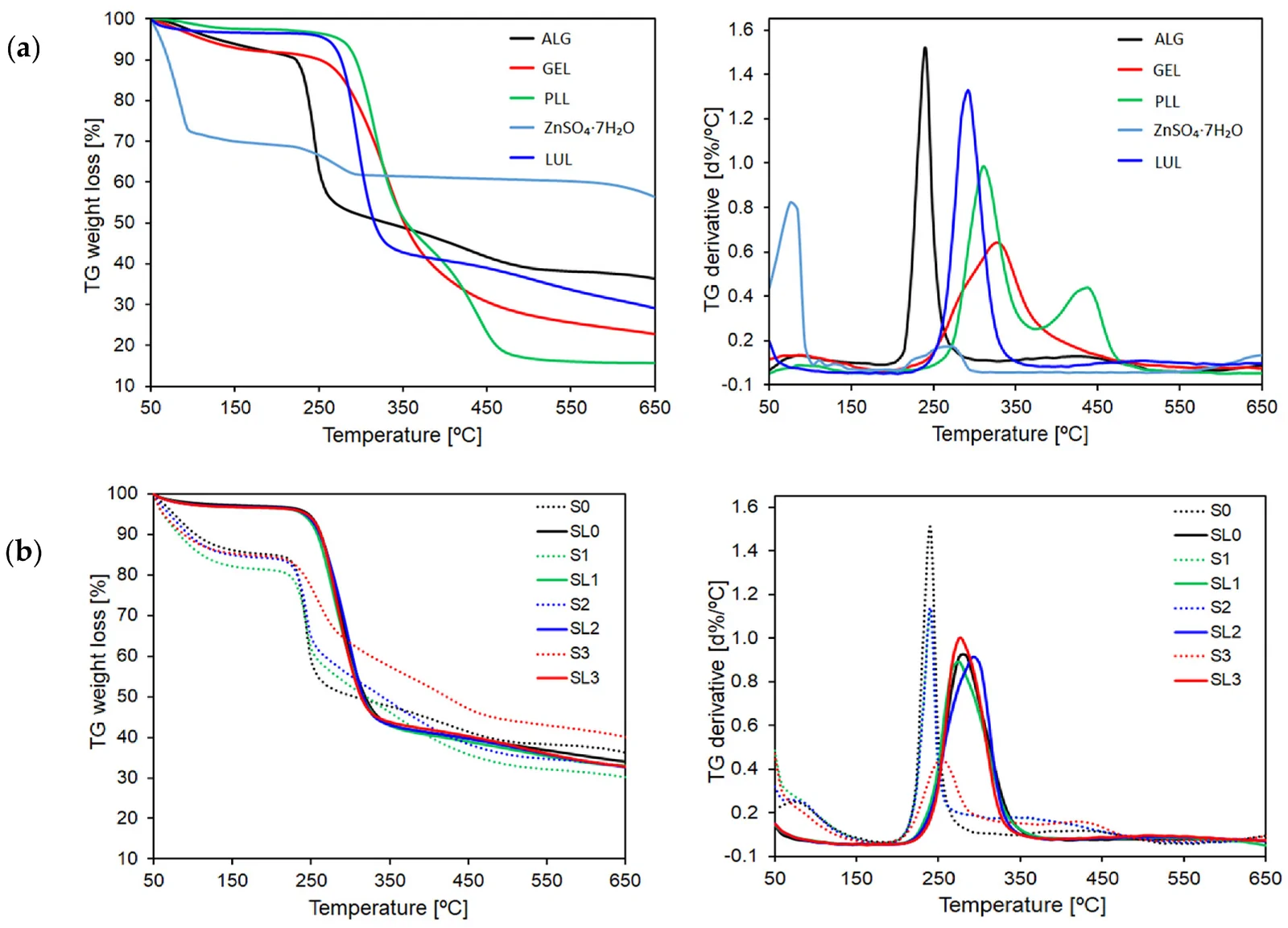
Thermogravimetric analysis: TGA and DTG curves of (**a**) the sponges’ individual components ALG, GEL, PLL, ZnSO_4_·7H_2_O, and LUL and (**b**) formulated sponges, shown as dotted lines (S0–S3, without drug) and solid lines (SL0–SL3, loaded with LUL).

Thermal analysis performed by thermogravimetric analysis (TGA) and differential scanning calorimetry (DSC) were employed to investigate the thermal stability and decomposition behavior of the prepared sponge formulations. Both the individual components and the composite sponges, with and without the drug, were analyzed to evaluate the influence of component interactions and drug incorporation on the thermal properties. TGA provides insights into the mass-loss patterns and decomposition steps of the materials, while DSC reveals thermal transitions and possible interactions between components [45]. Comparing formulations without drug (S0–S3) to their LUL-loaded counterparts (SL0–SL3) allows assessment of whether drug presence affect the thermal stability of the sponges (Figure 14). For the individual components (Figure 14a), ALG and GEL exhibited a minor weight loss below 150 °C, which might be attributed to the removal of moisture or residual solvents. Their major decomposition corresponded to polymer decomposition at 240 and 327 °C, respectively [46,47]. The thermogravimetric curve of PLL exhibited a two-step decomposition pattern. At first, main chain degradation and elimination of side chain amino groups occurred, with DTG (first derivative d%/°C) maxima at 310 and 440 °C [48]. ZnSO_4_·7H_2_O exhibited an initial mass loss below 100 °C, with a DTG maximum at approximately 76 °C, which can be attributed to the release of weakly bound and crystallized water. The resulting monohydrate remained thermally stable up to about 265 °C, above which the final water molecule was released. This is in agreement with the stepwise dehydration behavior reported for zinc sulphate hydrates in the literature [49,50]. LUL displayed thermal degradation starting around 230 °C, with a pronounced DTG peak at 293 °C. These results indicate distinct thermal stabilities and decomposition patterns for each component, which can influence the overall behavior of the sponges. For the sponge formulations (Figure 14b), the TG curves of drug-free formulations (S0–S3) exhibited a major mass-loss peak with a maximum at approximately 240 °C, which might be attributed to the thermal decomposition of the ALG polymer matrix. The LUL incorporation (SL0–SL3) led to slight modifications in the thermal decomposition profiles, manifested as a systematic shift of the DTG maxima by approximately 40 °C in all drug-loaded formulations. This behavior might indicate physical interactions and/or changes in the microenvironment of the polymer matrix induced by the presence of the drug. Importantly, the overall thermal stability of the sponges was maintained after drug loading, confirming the suitability of ALG/GEL matrices as carriers for LUL.

**Figure 15:**
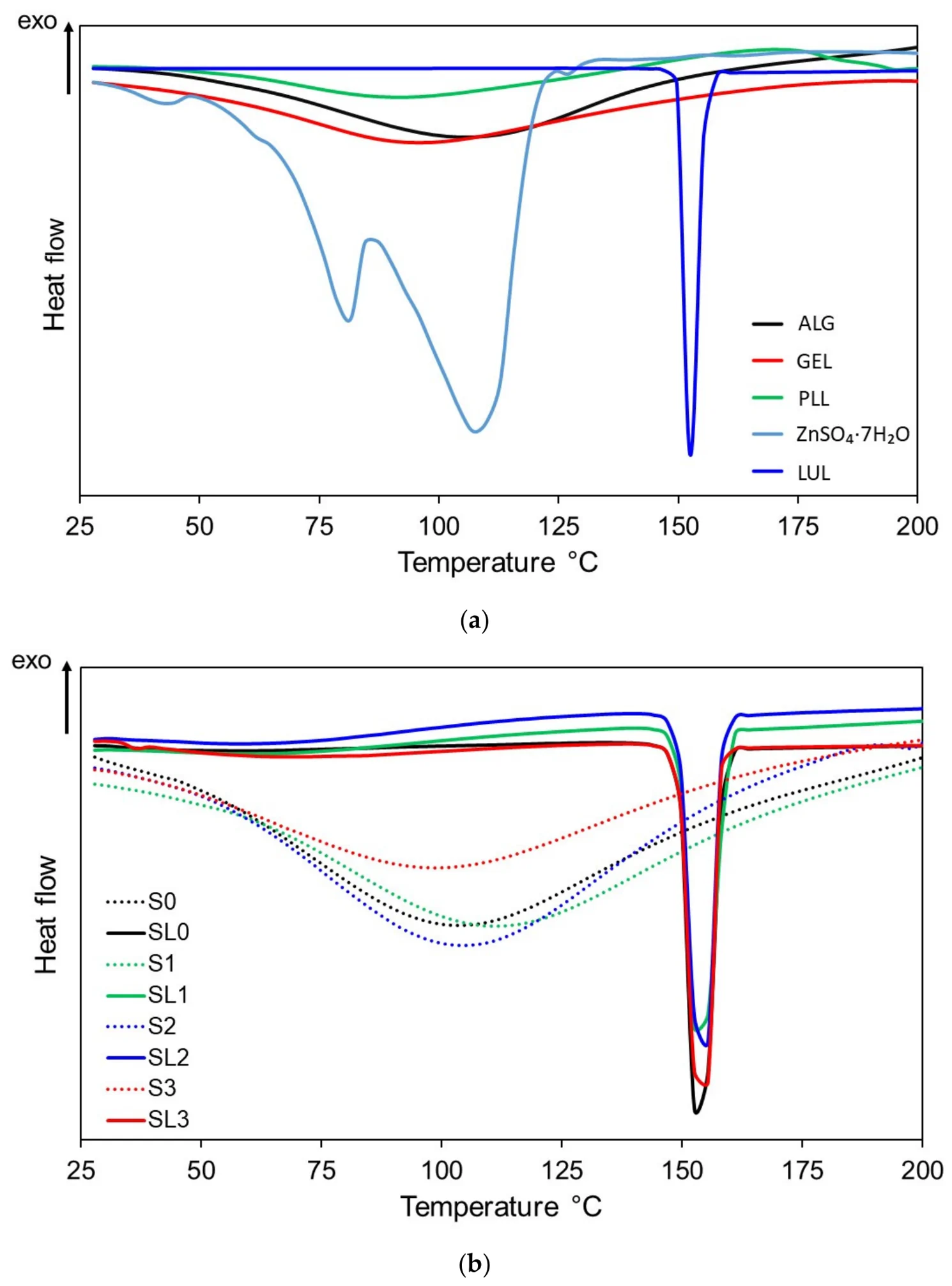
DSC curves of (**a**) individual components: ALG, GEL, PLL, ZnSO_4_·7H_2_O, and LUL; and (**b**) formulated sponges: dotted lines represent S0–S3 without LUL, and solid lines represent SL0–SL3 LUL-loaded formulations.

Analysis of thermograms of individual components obtained from the DSC method is expressed in Figure 15a. ALG, GEL, and PLL exhibited a broad endothermic transition peak in the temperature range of 50–170 °C, which is associated with the removal of physically bound water. In contrast, ZnSO_4_·7H_2_O exhibited a two-step dehydration process. This behavior was evidenced by two endothermic peaks at 80 and 107 °C, corresponding to the successive loss of water and consistent with the thermogravimetric data. LUL, on the other hand, exhibits a sharp melting peak at 153 °C, corresponding to the melting of the crystalline drug [51]. Figure 15b presents the DSC thermograms of sponges without LUL (dotted lines) and with LUL (solid lines). The drug-free sponges exhibited only thermal transitions related to dehydration, manifested as broad endothermic peaks analogous to those observed for the individual polymeric components. No distinct melting or decomposition peaks were detected within the investigated temperature range. In contrast, the thermograms of the LUL-loaded formulations were dominated by a well-defined endothermic peak at approximately 153 °C, corresponding to the melting of the encapsulated drug. The presence of this peak confirmed successful incorporation of LUL within the sponge matrix and indicates that the drug retains its crystalline character after encapsulation, although partial physical interactions with the polymeric matrix cannot be excluded.

#### 3.2.6. Thermal Analysis and Attenuated Total Reflectance–Fourier Transform Infrared Spectroscopy Determination

**Figure 16:**
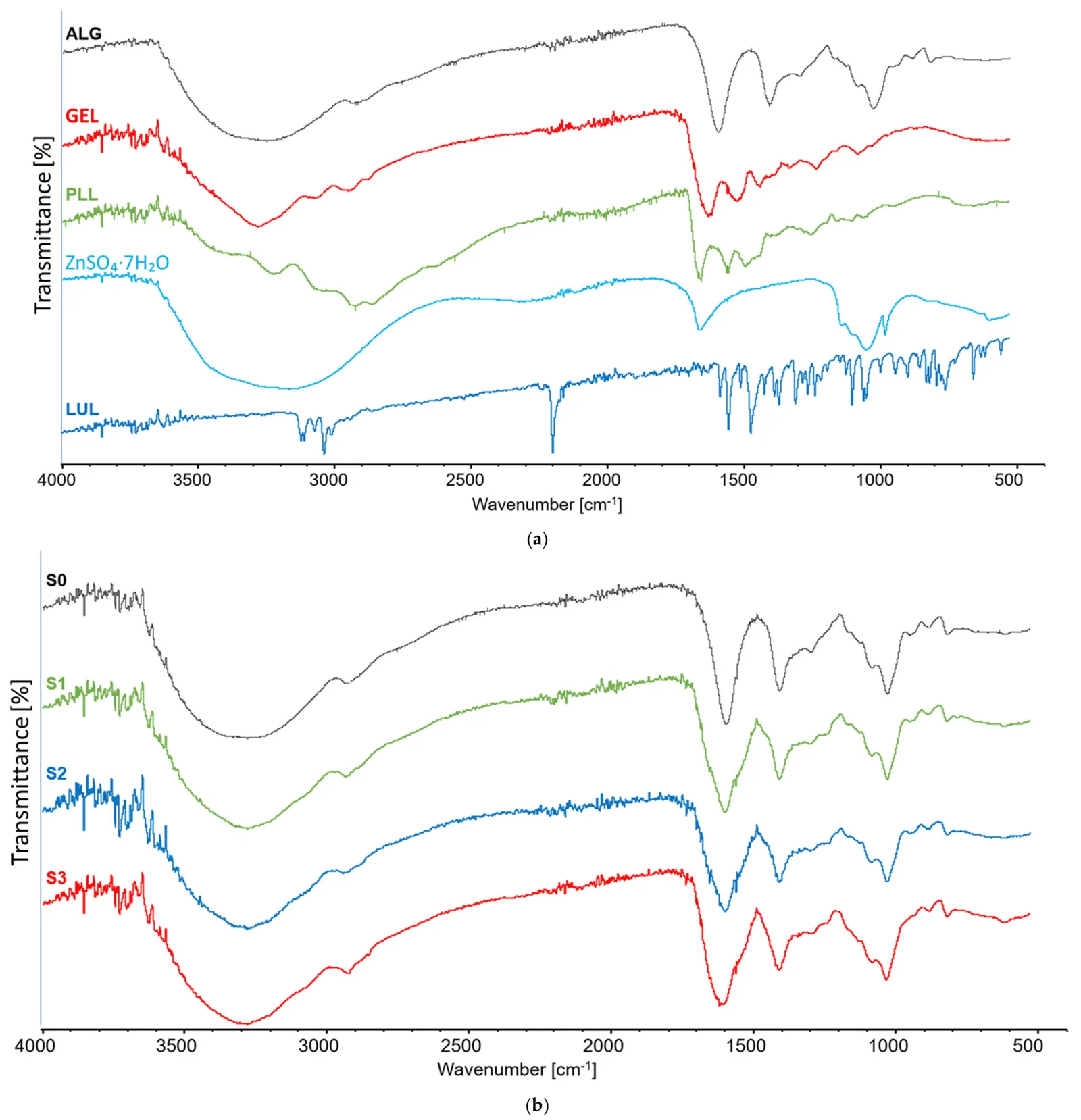

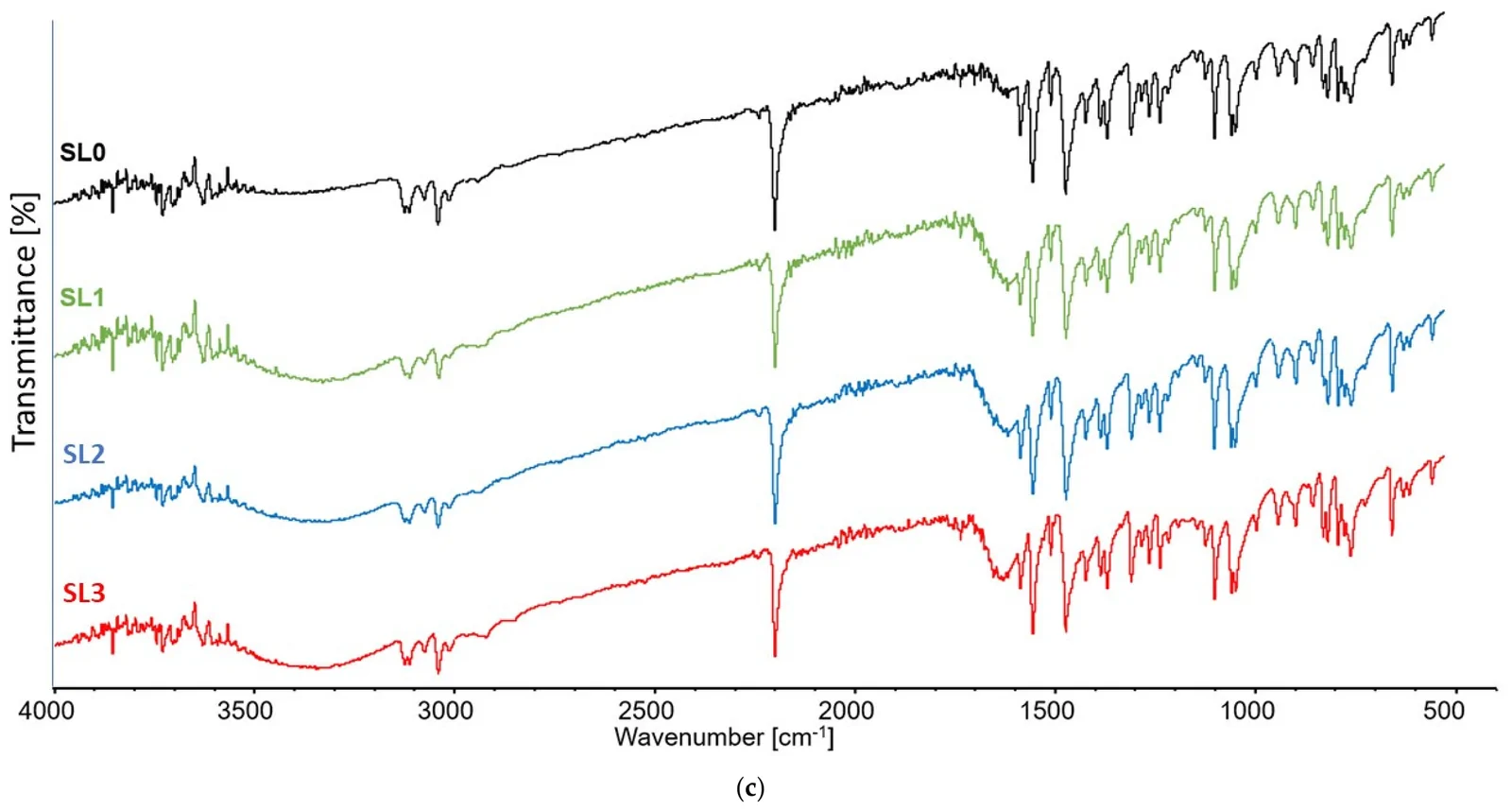
Infrared spectra of (**a**) individual components: ALG, GEL, PLL, ZnSO_4_·7H_2_O, and LUL; (**b**) formulated sponges without LUL and (**c**) LUL-loaded formulations.

FT-IR spectroscopy was applied in order to determine the individual components and the resulting sponge formulations and to evaluate possible changes in their spectral features after drug incorporation. The infrared spectra of the raw materials (ALG, GEL, PLL, ZnSO_4_·7H_2_O, and LUL) (Figure 16a), the formulated sponges without LUL (Figure 16b), and the LUL-loaded formulations (Figure 16c) were evaluated. In the spectrogram of pure ALG, a broad band responding to the stretching vibrations of -O-H bonds was noted, characterized by a maximum at 3251 cm^−1^, as well as a band at 2928 cm^−1^ associated with the stretching vibrations of -C-H bonds. Moreover, asymmetric (1593 cm^−1^) and symmetric (1405 cm^−1^) vibrations of the -COO- carboxyl groups were identified. The spectrum also presented pyran ring at 1025 cm^−1^ with stretching vibrations of the C-O bonds. In the formulation S0 spectra, all signs characteristic of ALG were present. However, they were shifted toward the blue end of the spectrum, with bands originating from -O-H bond vibrations by approximately 30 cm^−1^, while the remaining bands were shifted by about 3–4 cm^−1^. All bands characteristic of ALG were observed in the spectrum of sample S1. The peak corresponding to the vibrations of the carboxylate groups shifted slightly from 1593 to 1598 cm^−1^ and broadened significantly (an increase in the full width at half maximum—FWHM—from 81 to 136 cm^−1^). These phenomena resulted from the overlap of the vibrations of the asymmetric ALG carboxylate groups with the amide I vibrations of GEL, as well as from changes in the chemical environment of the functional groups due to strong interchain interactions (including electrostatic interactions –COO^−^···NH_3_^+^). Consequently, the amide I (~1630 cm^−1^) and amide II (~1520 cm^−1^) bands undergo partial quenching or merge with the carboxylate band, forming a single broad spectral envelope. At the same time, the amide III band (~1233 cm^−1^) remained clearly visible, confirming the presence and preservation of the protein structure of GEL in a sponge-like matrix. In the spectra of the double cross-linked (S2) and triple cross-linked (S3) samples, no characteristic band peaks were observed for any of the cross-linking agents. This may be due to their low concentration in the mixture, which is below the detection limit of the ATR-FTIR method (0.025% and 0.2%, respectively). Nevertheless, in the spectra of the S2 and S3 preparations, the O-H stretching bands originating from ALG underwent a slight blue shift, which might indicate the formation of polyelectrolyte complexes between PLL and ALG [52]. An increase in FWHM was also observed for the S2 formulation (FWHM = 132 cm^−1^) and for the S3 formulation (FWHM = 136 cm^−1^). This probably indicated the presence of the amide band A and was the result of the influence of stretching and bending vibrations of the N–H bonds, as well as stretching vibrations of the C–O and C–N double bonds of the amide group in PLL [53,54]. In contrast, the effect of Zn^2+^ ions appeared to be more pronounced, particularly in the region around 1600 cm^−1^, where a shift of the peak toward higher wavenumbers (from 1598 to 1609 cm^−1^) was observed in sample S3. This fact may indicate a change in the environment of the ALG carboxylate groups as a result of interactions with Zn^2+^ ions. Furthermore, in the spectra containing active substance (SL0–SL3), subtle signals associated with LUL-specific bond vibrations were detected. The LUL FTIR spectrum exhibited a characteristic band at 2201 cm^−1^, corresponding to the stretching vibrations of the nitrile group (C≡N). In the range of 3114–3011 cm^−1^, stretching vibrations of C–H bonds in aromatic systems were observed. The bands at 1587 and 1556 cm^−1^ were associated with skeletal vibrations of aromatic rings and C=N groups present in the imidazole ring. Bands in the 1284–1239 cm^−1^ and 1101–1049 cm^−1^ ranges might be attributed to C–N and C–S–C vibrations, respectively, while the band at 658 cm^−1^ is characteristic of the C–Cl bond. Furthermore, in the IR spectra of formulations containing LUL (SL0–SL3), subtle bands associated with vibrations characteristic of LUL were detected. A nitrile group (C≡N) band was present at approximately 2200 cm^−1^, confirming the presence of the drug in the matrix. Compared to pure LUL, an additional broad band appeared at approximately 3311 cm^−1^, associated with the stretching vibrations of the O–H and N–H groups of the polymeric components, as well as a band at 1617 cm^−1^, attributed to the vibrations of carboxylate and/or amide groups. The absence of significant shifts in the C≡N band suggests that LUL has been physically encapsulated in the matrix without the formation of new chemical bonds.

#### 3.2.7. Antifungal Activity

**Figure 17:**
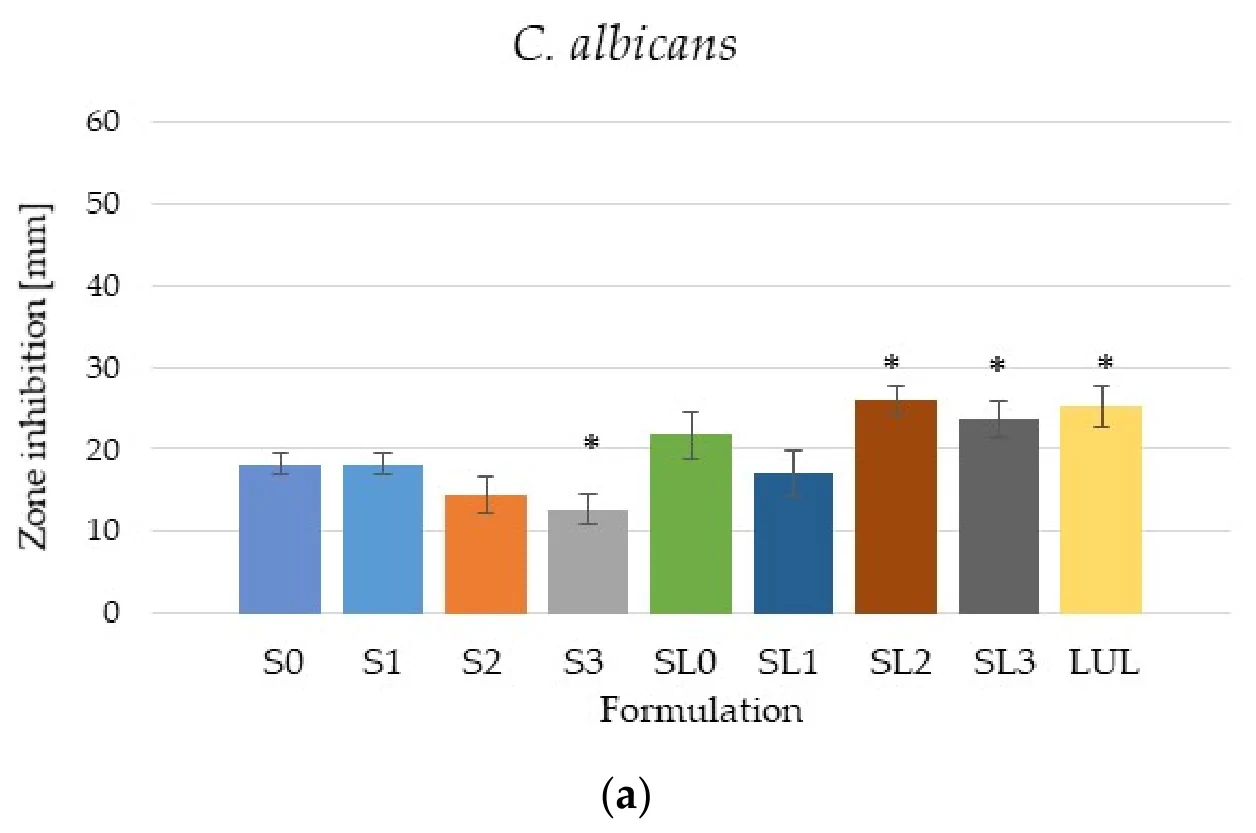

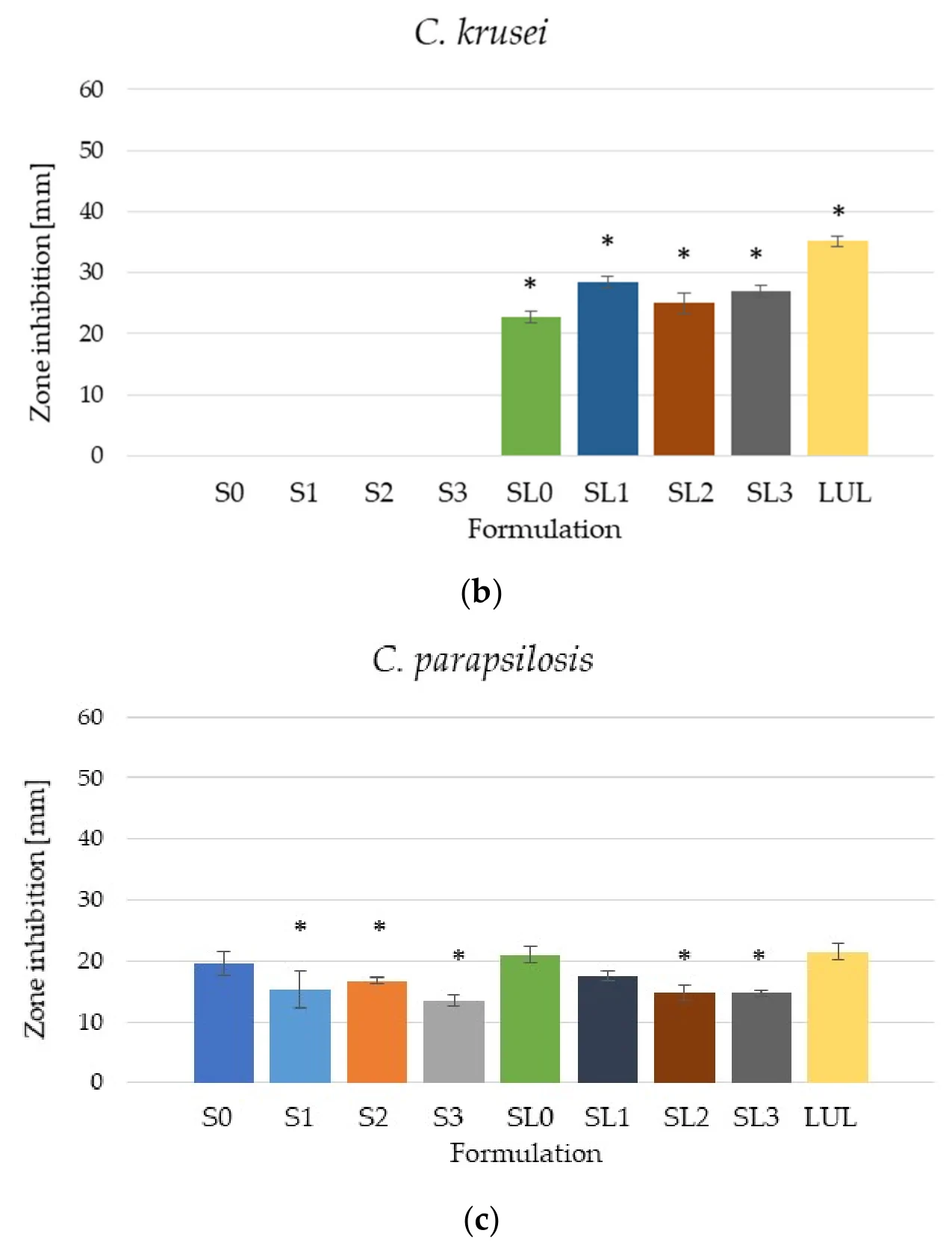
Antifungal effect of sponge placebo (S0–S3), LUL-loaded formulations (SL0–SL3), and LUL/DMSO solution (LUL) applied and control for (**a**) *C. albicans*, (**b**) *C. krusei*, and (**c**) *C. parapsilosis* (mean ± SD, *n* = 6, * significant statistical difference (*p* < 0.05) versus correspondent value of S0).

**Figure 18:**
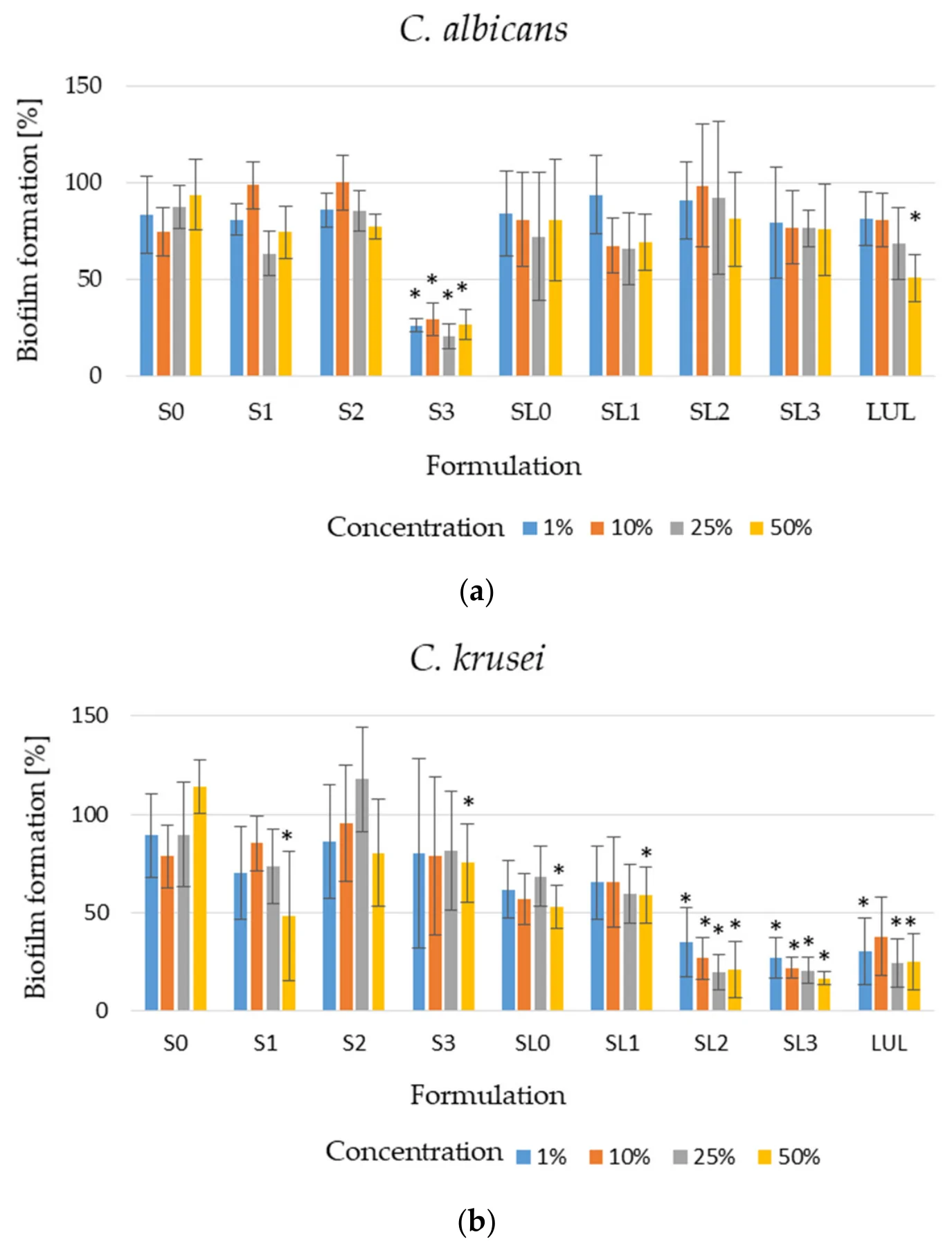

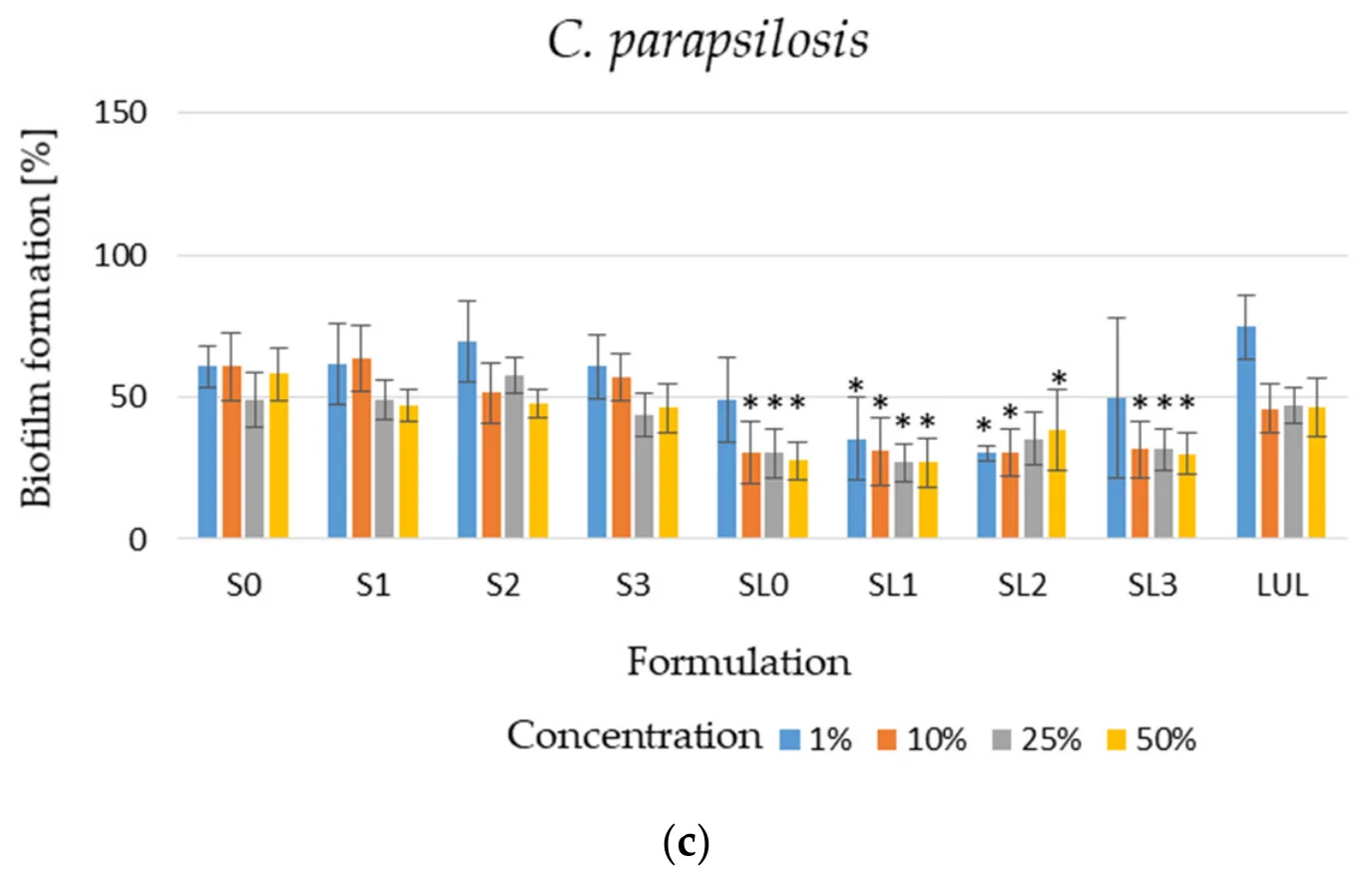
Formation of (**a**) *C. albicans*, (**b**) *C. krusei*, and (**c**) *C. parapsilosis* biofilm in the presence of tested formulations without LUL (S0–S3), with LUL (SL0–SL3), 1% LUL solution in DMSO, and PBS (as control) (mean ± SD, *n* = 6, * significant statistical difference (*p* < 0.05) versus to correspondent value of S0).

The evaluation of the antifungal activity of vaginal drug dosage forms is essential for assessing their efficacy against pathogens responsible for intimate infections, particularly fungi of the genus Candida. This analysis also enables the determination of the formulation’s impact on the rate and efficiency of active ingredient release and to predict the therapeutic efficacy of the preparation under vaginal conditions. The antifungal activity of the tested formulations was evaluated with the diffusion disk method, which determines the sponges ability to inhibit microbial growth. Additionally, an analysis was performed to assess the effect of the tested samples on the formation of fungal biofilm. The results obtained served as the basis for comparing the antifungal properties of the designed formulations. It was found that all sponges containing the active ingredient exhibited antifungal activity (Figure 17 and Figure 18). In the instance of a LUL-loaded formulation, the highest antifungal activity was recorded in the case of *Candida krusei* strains and the zones of growth inhibition varied from 22.75 ± 0.96 mm in formulation SL0 to 28.50 ± 1.73 mm in formulation SL1 (Figure 17b). With regard to *Candida albicans* strains, the highest zones of growth inhibition were noted for formulation S2 (26.00 ± 2.16 mm), while the minimum was noted for formulation SL1 (17.00 ± 1.83 mm). The remaining formulations exhibited activity comparable to that of the active ingredient alone (Figure 17a). In the case of *Candida parapsilosis*, the non-cross-linked formulation expressed the most activity (19.50 ± 3.0 mm). In the case of LUL-loaded formulations, antifungal activity dependent on the degree of cross-linking was observed (Figure 17c). During analysis of *C. albicans* species, it was found that non cross-linked SL0 and cross-linked SL1 formulations possessed similar zones of growth inhibition (18.25 ± 1.26 mm and 18.20 ± 2.17 mm, respectively). In contrast, formulation SL2 and SL3 demonstrated a decrease in antifungal activity to 14.40 ± 1.82 mm and 12.67 ± 1.51 mm, respectively. However, in the case of *Candida parapsilosis*, a decrease in activity was noted in all cross-linked formulations compared to the unmodified formulation. An interesting phenomenon was the antifungal activity observed even in the placebo controls against both *Candida albicans* and *Candida parapsilosis* (Figure 17a,c). The antifungal activity of the placebo formulation is mainly attributable to the antifungal qualities of ALG per se. ALG forms a viscous hydrogel layer on the surface of microbial cells, limiting nutrient absorption, compromising cell membrane integrity, and reducing microbial adhesion and biofilm formation. Furthermore, negatively charged ALG chains might interact with external cellular structures, potentially increasing membrane permeability and promoting the leakage of intracellular components [55,56].

The effect of the prepared sponges on antifungal efficacy depended on the susceptibility of the fungal strain and the type of cross-linking agent. As cross-linking agents, substances with documented antifungal properties or that enhance the antifungal effect were used. GEL might enhance antifungal performance through viscosity modulation and the formation of a gel-like barrier which can restrict fungal proliferation and interfere with microbial adhesion and biofilm development [19]. It was noticed that GEL presence in the formulation significantly increased antifungal activity against *Candida krusei*, which expressed in formulation SL1 (28.50 ± 1.73 mm). Due to the presence of positively charged amino groups, PLL exhibits a broad spectrum of antimicrobial activity against bacteria, fungi, and viruses. Its activity is primarily based on electrostatic interactions with the cell membrane of microorganisms, leading to disruption of its integrity, loss of cellular components, and cell death. Additionally, PLL induces the formation of reactive oxygen species (ROS) and disrupts cellular metabolic processes [57]. The PLL addition increased antifungal activity in the case of *C. albicans* (26.00 ± 2.16 mm) and *C. krusei* (25.00 ± 1.00 mm). The mechanism of Zn^2+^ antifungal activity is primarily linked to the disruption of the integrity of fungal cell membranes and cell walls, which leads to increased permeability and the loss of intracellular components. Zn^2+^ ions can also inhibit enzyme activity and key cellular metabolic processes. Additionally, Zn^2+^ ions induces oxidative stress by ROS generating, leading to cellular damage and fungal cell death [58]. The presence of Zn^2+^ ions improved antifungal activity against the strains *Candida albicans* (23.67 ± 1.53 mm) and *Candida krusei* (27.00 ± 1.00 mm).

In summary, for *Candida albicans* and *Candida krusei*, the addition of cross-linking agents—GEL, PLL, and Zn^2+^—led to an increase in the antifungal activity of the sponge formulations, despite the formation of a denser network structure, reduced swelling, and prolonged drug release. Interestingly, in the case of *Candida parapsilosis*, no synergistic effect of cross-linking agents with antifungal activity was observed, whereas an increase in the degree of cross-linking weakened this effect, which may result from the strain’s different sensitivity to the substances employed.

A Candida biofilm is an organized structure of cells embedded in an extracellular matrix, adhering to host tissues or the surfaces of biomaterials. By creating a stable microenvironment, the biofilm protects the cells from adverse external factors, such as drying out, changes in pH, temperature fluctuations, or exposure to chemicals. Both its complex architecture and the altered metabolic activity of the microorganisms contribute to increased resistance to antimicrobial drugs, hindering the effective eradication of infections. Research on Candida biofilm is crucial for the development of new therapies capable of penetrating this structure and effectively treating chronic infections [59]. Analysis of the prepared sponges revealed that all formulations—both with LUL and the placebo—exhibited anti-film formation activity against the tested strains (Figure 18). An evaluation of the placebo formulations confirmed that all variants exhibited anti-biofilm activity, although the intensity of this activity varied depending on the Candida strain and the sponges’ composition. In the case of *C. albicans*, the highest anti-biofilm activity was exhibited by formulation S3, suggesting that its composition or degree of cross-linking, as well as the presence of Zn^2+^ ions, contributed to the inhibition of biofilm formation (Figure 18a). In the study of the *C. krusei* strain, it was noticed that the formulations cross-linked by GEL and triple cross-linked by Zn^2+^ ions demonstrated anti-biofilm activity (Figure 18b). For formulations S0 and S2, no inhibitory effect was observed; the rate of biofilm formation reached values close to 100%. Against *C. parapsilosis*, all placebo formulations possessed high film-inhibiting properties among the analyzed strains (Figure 18c), but no difference in activity between the formulations was observed. Significant results were obtained for the cross-linked formulations at a 50% concentration, indicating that a higher polymer network density has a beneficial effect on reducing colonization. In the case of the drug-loaded formulation, it was demonstrated that, against the tested species *Candida albicans*, formulations containing LUL exhibited activity comparable to the pure drug, indicating that the incorporation of the active ingredient into the sponge did not reduce its efficacy (Figure 18a). It may be assumed that the active substance was effectively released from the matrix and retained its activity against biofilm cells. In the case of *C. parapsilosis*, the anti-biofilm effect was independent of the degree of cross-linking, but the activity of the formulations was higher than that of the pure drug (Figure 18c). For the *Candida krusei* strain, a distinct difference was observed (Figure 18b). Formulations SL2 and SL3 exhibited the highest anti-biofilm activity against *C. krusei*, exceeding the efficacy of the drug alone, which may indicate a synergistic effect of the components resulting from the combined PLL and Zn^2+^ ions action. Possible mechanism responsible for the increased efficacy is the facilitated diffusion of Zn^2+^ ions and the drug within the biofilm [60]. PLL, as a polycationic polymer, may promote the penetration of these components into deeper layers of the biofilm structure. Additionally, the observed effect may result from disruption of the extracellular matrix organization through Zn^2+^ ions’ influence on the structure of polysaccharides and electrostatic destabilization induced by PLL [20]. The observed synergistic effect may result from the combined action of both components on the metabolism of biofilm cells, including the inhibition of Zn^2+^-dependent enzyme action and the disruption of cell membrane integrity by PLL, which leads to a more pronounced reduction in cellular metabolic activity than the pure drug [61,62]. Improved effectiveness of the formulation results from modifications to the sponges’ properties, where an appropriate degree of cross-linking promotes the effective release of active ingredients and their prolonged interaction with the biofilm.

#### 3.2.8. Cytotoxicity Assay

**Figure 19:**
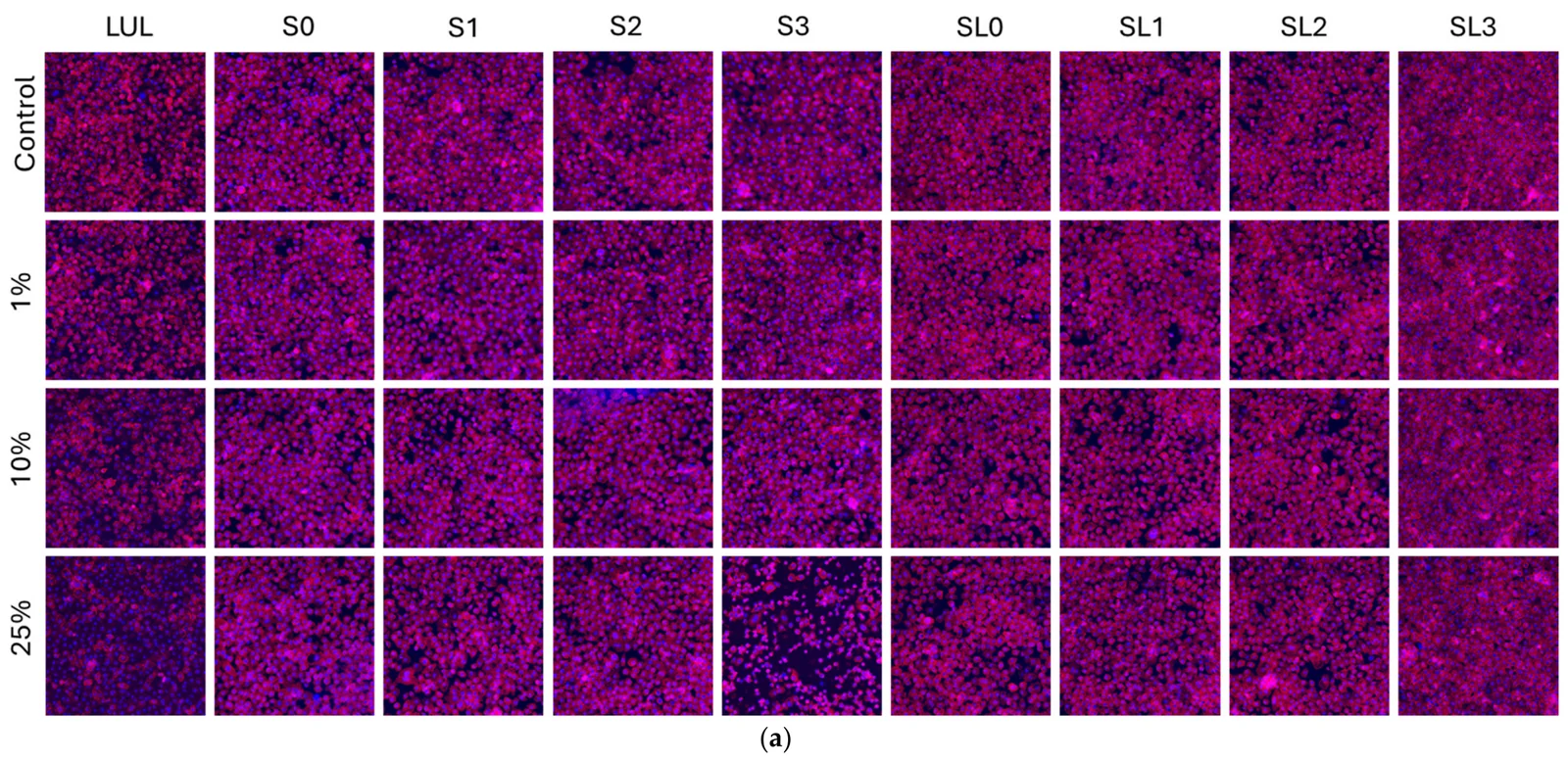

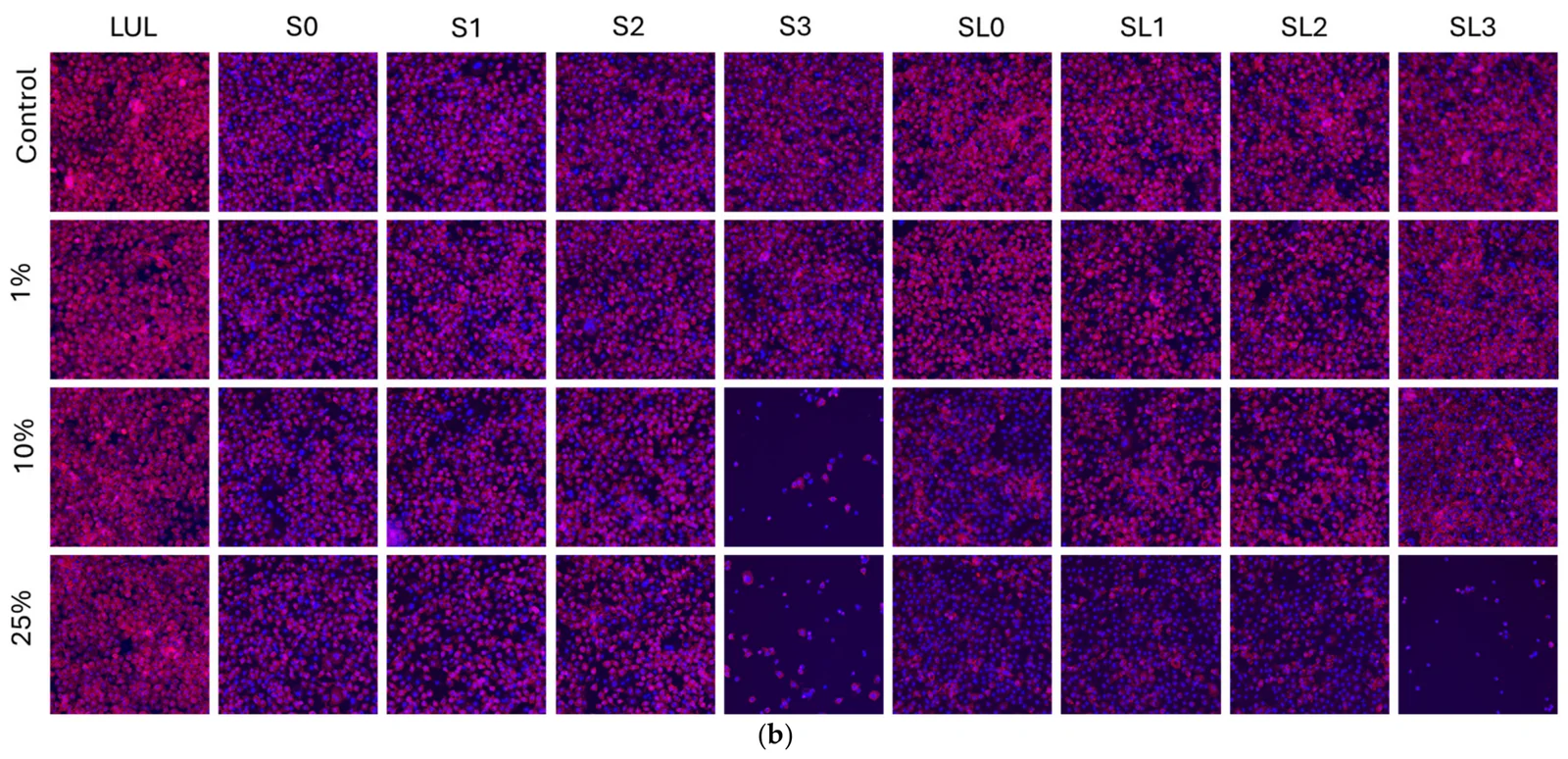
The morphology of VK2 E6/E7 prepared from tested formulations without LUL (S0–S3), with LUL (SL0–SL3), LUL solution (LUL) (as control) upon (**a**) 4 h and (**b**) 24 h exposure to extracts. Extracts were diluted to the final concentrations of 1%, 10%, and 25%; pink and blue colors indicate the cytoskeleton and nuclei of treated cells.

**Figure 20:**
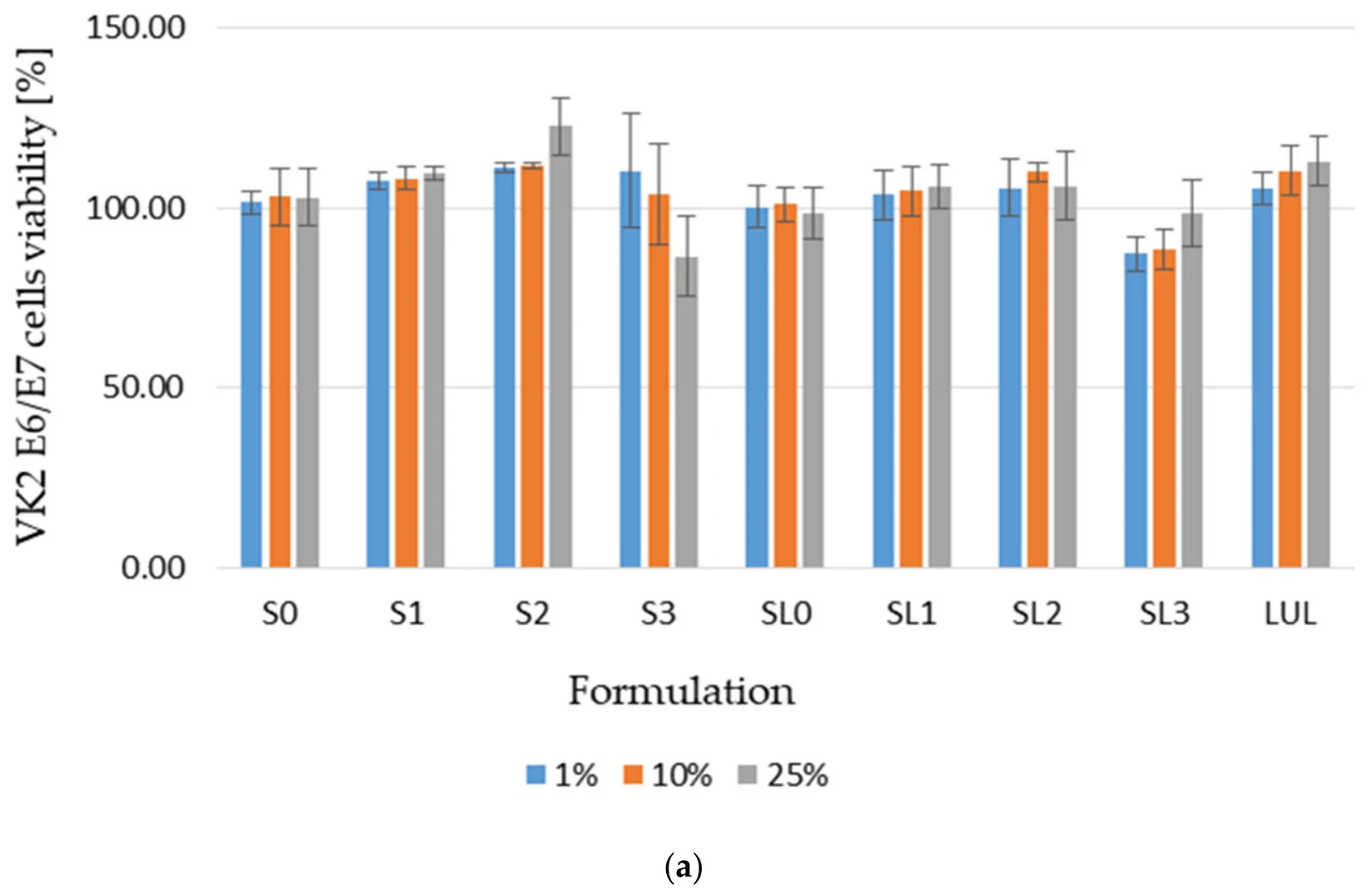

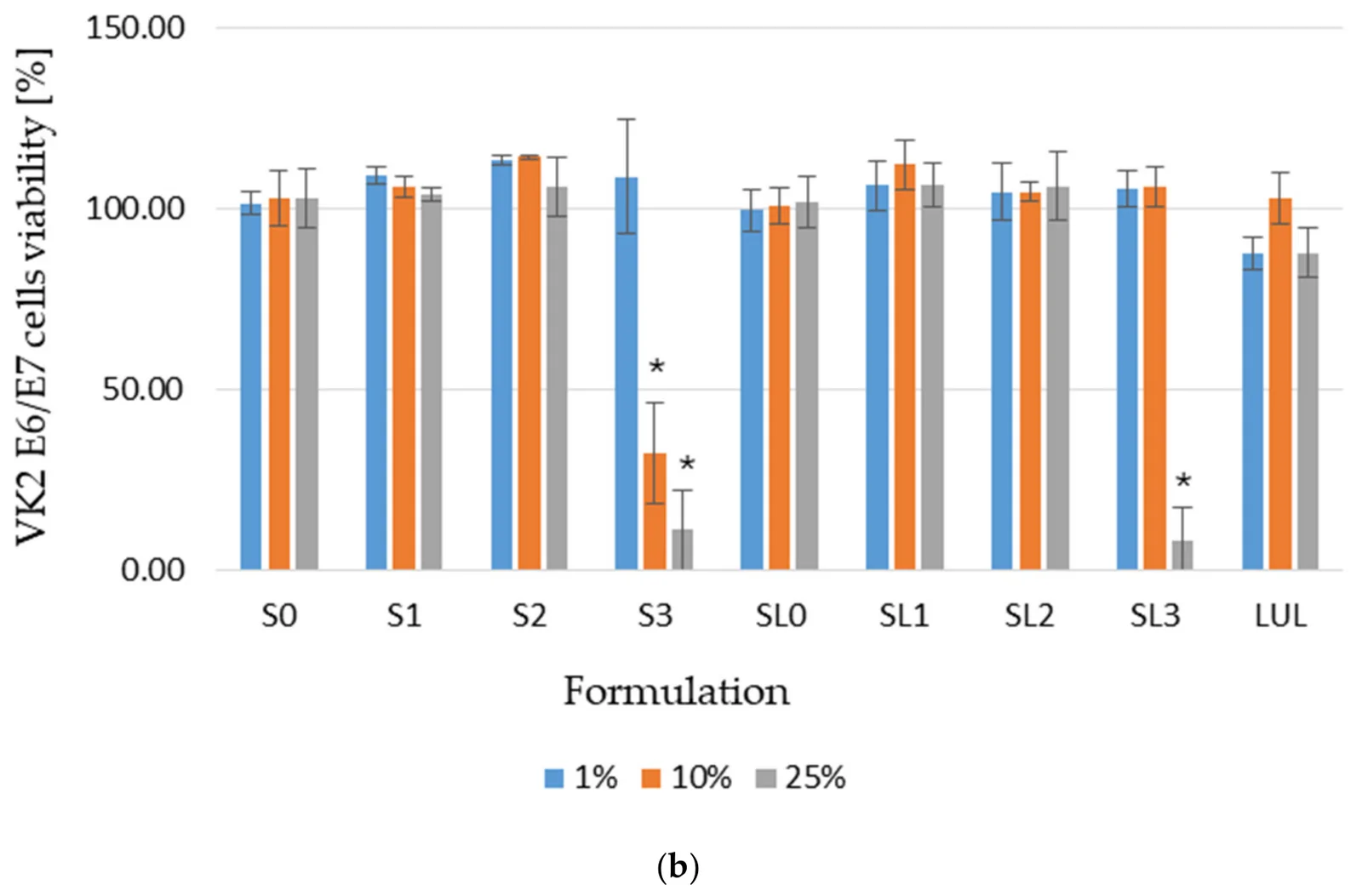
The viability assessed using MTT test of VK2 E6/E7 exposure to extracts prepared from tested formulations without LUL (S0–S3), with LUL (SL0–SL3), and LUL solution (LUL) (as control) upon (**a**) 4 h and (**b**) 24 h. Extracts were diluted to the final concentrations of 1%, 10% and 25%. LUL solution in DMSO and PBS was applied as control. Results are presented as mean ± SD, *n* = 3, * significant statistical difference (*p* < 0.05) versus to correspondent value of S0).

The MTT assay is one of the most commonly employed methods for assessing cellular metabolic activity and viability, based on the ability of mitochondrial dehydrogenases to reduce the reduction of 3-(4,5-dimethylthiazol-2-yl)-2,5-diphenyltetrazolium bromide (MTT) to insoluble formazan. The amount of the resulting product, measured spectrophotometrically after dissolution, is proportional to the metabolic activity of the cells; therefore, this test is widely used in cytotoxicity studies [63]. The MTT assay utilizing the human vaginal epithelial cell line VK2 E6/E7 is a key tool for assessing cell viability following exposure to test substances and formulations, enabling the determination of their cytotoxicity under in vitro conditions. The conducted studies proved that all tested formulations demonstrated high biocompatibility with vaginal epithelial cells. The primary criteria for assessing biocompatibility were the preservation of normal cell morphology and the absence of significant changes in cell count following exposure to the tested formulations. As shown in Figure 19 and Figure 20, no significant morphological changes were noted in the cells exposed to the tested formulations after 4 h of incubation. The cells retained their characteristic spindle-shaped morphology and strong adhesion to the culture surface, indicating the maintenance of a normal physiological state. Additional confirmation of biocompatibility was provided by the presence of fluorescence characteristic of living cells and the absence of distinct changes in cellular organization observed in the microphotographs (Figure 19). Although slight cell contraction was observed in some variants, the images obtained did not indicate the presence of a cytotoxic effect on the tested cell line. To quantitatively assess this phenomenon, an analysis of the number of cell nuclei was conducted based on microphotographs taken of all tested samples. This analysis did not reveal a significant reduction in the number of nuclei after 4 h of exposure to the tested formulations (Figure 20a). The results of the cell viability assays confirmed that none of the formulations exhibited cytotoxic effects on vaginal epithelial cells. Both the placebo and LUL-containing formulations maintained cell viability levels close to 100% after 4 h and 24 h of incubation at a 1% concentration (Figure 20a,b). For higher concentrations of the tested formulations, cell viability remained comparable to that observed for the active ingredient alone, indicating that the carriers used did not exacerbate its potential cytotoxic effect. Formulation S3 contained Zn^2+^ ions, for which a concentration-dependent decrease in cell viability was observed. After 24 h, viability decreased from 108.92 ± 2.17% at a 1% concentration to approximately 32.37 ± 2.99% at a 10% concentration, reaching 10.90 ± 1.34% at a 25% concentration. A reduction in viability (7.88 ± 0.41%) after 24 h was also noted in the case of the formulation SL3, but only at a concentration of 25%. This was confirmed by images of the morphology of VK2 E6/E7 (Figure 20b). The adverse effects of Zn^2+^ ions in formulation S3 at a concentration of 10% were manifested by morphological changes in epithelial cells, such as loss of their characteristic elongated shape, cell shrinkage, and a decrease in cell number. A decrease in the cell number and the appearance of rounded cells detaching from the culture surface, which is characteristic of cell death processes, can also be observed. As the 25% concentration of formulation S3 and SL3, a cytotoxic effect was noted, characterized by a reduction in cell viability. This phenomenon might be explained by the known effect of Zn^2+^ ions on the functioning of epithelial cells. Zinc is an element essential for the proper course of cellular processes, such as DNA synthesis, proliferation, and protection against oxidative stress [60]. Elevated levels concentrations of Zn^2+^ ions might lead to disturbances in cellular homeostasis, mitochondrial damage, increased production of reactive oxygen species, and the induction of apoptosis, resulting in reduced cell viability. However, this effect is highly dependent on concentration, exposure time, and cell type [64]. Studies by Sharif et al. expressed that excess zinc might exert a cytotoxic effect on human oral keratinocytes, leading to disruption of their biological functions. At the same time, the authors concluded that maintaining zinc concentrations in the range of 4–16 μM is necessary to limit DNA damage and maintain genome stability in cultured human keratinocytes [65]. Studies by Sales et al. conducted on human HaCaT keratinocytes showed that the effect of ZnCl_2_ is dependent on the concentration and time of exposure. Cytotoxicity was observed at concentrations of ≥500 µg/mL after 3 h of incubation, ≥18 µg/mL after 12 h, and ≥14 µg/mL after 24 h, indicating an increasing toxic effect with prolonged exposure. Concentrations of 1–5 µg/mL did not affect cell proliferation after 72 h, and concentrations up to 10 µg/mL did not show significant cytotoxicity. Furthermore, ZnCl_2_ induced the expression of genes related to antioxidant response and glycoprotein synthesis, suggesting potential protective and regenerative properties at low concentrations [66].

In summary, all tested formulations—including the 1% placebo formulation, formulations containing the active ingredient, and LUL solution—did not significantly reduce cell viability after 24 h of incubation. Furthermore, at 10% and 25% concentrations, all tested formulations, except S3 and SL3, demonstrated biocompatibility with VK2 E6/E7 cells. These results fulfill the biocompatibility requirements defined in ISO 10993 and the FDA guidelines, which state that a material is nontoxic if in vitro cell viability exceeds 70% after 24 h of exposure [67].

## 4. Conclusions

The results received from this study demonstrated that it is possible to obtain sponges based on triple cross-linked ALG with LUL by lyophilization technique. A triple cross-linking process was developed by applying two complementary cross-linking mechanisms: electrostatic cross-linking with positively charged substances—0.75% GEL and 0.1% PLL—and ionic cross-linking involving 3% zinc sulphate heptahydrate. It was shown that modification of ALG involving triple cross-linking by GEL, PLL, and Zn^2+^ led to an increase in mechanical properties, improved mucoadhesive properties toward the vaginal mucosa, and an extension of the active substance release profile to 3 h. In addition, the cross-linking process contributed to the improved antifungal activity against *Candida albicans*, *Candida krusei*, and *Candida parapsilosis*. The triple cross-linked formulation SL3 formulation demonstrated significant ability to inhibit biofilm formation by *Candida* species, especially *Candida krusei.* Biocompatibility studies confirmed that the prepared triple cross-linked formulations were biocompatible with the human vaginal VK2 E6/E7 epithelial cell line, depending on the exposure time and applied concentration. The developed sponges might constitute an encouraging drug formulation for intravaginal LUL delivery.

## Figures and Tables

**Table 1:** Composition of ALG non cross-linked, ALG/GEL single cross-linked, ALG/GEL/PLL dual cross-linked, and ALG/GEL/PLL/Zn^2+^ triple cross-linked hydrogel formulations without active substance (S0–S3) and with LUL (SL0–SL3).

Formulation	ALG (g)	GEL (g)	PLL (g)	Zn^2+^ (g)	LUL (g)	Water (Up To)
S0	1.0	–	–	–	–	100.0
S1	1.0	0.375	–	–	–	100.0
S2	1.0	0.375	0.025	–	–	100.0
S3	1.0	0.375	0.025	0.3	–	100.0
SL0	1.0	–	–	–	5.0	100.0
SL1	1.0	0.375	–	–	5.0	100.0
SL2	1.0	0.375	0.025	–	5.0	100.0
SL3	1.0	0.375	0.025	0.3	5.0	100.0

**Table 2:** Viscosity and pH values of designed non cross-linked hydrogels, single cross-linked with GEL, dual cross-linked with GEL and PLL, and triple cross-linked with Zn^2+^ ions with different volumes of cross-linking agent (mean ± SD, *n* = 3).

Formulation	pH	Viscosity (mPa∙s)
ALG	9.10 ± 0.02	259.67 ± 48.78
ALG/GEL	8.64 ± 0.05 *	496.10 ± 70.76
ALG/GEL/PLL	8.69 ± 0.01 *	620.13 ± 49.61
ALG/GEL/PLL/Zn 2.5 2.52.5	7.98 ± 0.05 *	476.26 ± 44.37
ALG/GEL/PLL/Zn 5	7.73 ± 0.02 *	818.57 ± 95.00 *
ALG/GEL/PLL/Zn 10	7.62 ± 0.02 *	12,435.57 ± 509.16 *
ALG/GEL/PLL/Zn 15	7.48 ± 0.03 *	12,080.04 ± 307.15 *

* significant statistical difference (*p* < 0.05) versus correspondent value of ALG.

**Table 3:** Features of developed placebo sponges (formulations S0–S3) and those containing LUL (formulations SL0–SL3) (mean ± SD, *n* = 3).

Formulation	Mean Weight (mg)	Mean Diameter (mm)	Mean Length (mm)	Drug Content (%)
S0	9.46 ± 0.21	8.13 ± 0.11	20.19 ± 0.10	–
S1	12.76 ± 0.21 *	8.17 ± 0.11	20.22 ± 0.13	–
S2	12.61 ± 0.66 *	8.19 ± 0.12	20.20 ± 0.11	–
S3	12.93 ± 0.16 *	8.15 ± 0.09	20.34 ± 0.24	–
SL0	112.68 ± 3.05 *	8.15 ± 0.06	20.13 ± 0.07	105.82 ± 0.78
SL1	114.19 ± 1.37 *	8.10 ± 0.06	20.25 ± 0.14	99.76 ± 0.19
SL2	113.30 ± 3.19 *	8.13 ± 0.10	20.19 ± 0.14	100.84 ± 0.21
SL3	114.73 ± 3.82 *	8.14 ± 0.10	20.25 ± 0.15	104.43 ± 0.62

* significant statistical difference (*p* < 0.05) versus to correspondent value of S0.

**Table 4:** Models of LUL release from sponge formulations SL0–SL3.

Formu- Lation	Zero-Order Kinetics		First-Order Kinetics		Higuchi Model		Korsmeyer-Peppas Model			Hixson Model	
	R^2^	K	R^2^	K	R^2^	K	R^2^	K	*n*	R^2^	K
SL0	0.80	9.71	0.76	0.10	0.85	27.25	0.82	0.32	0.12	0.62	1.21
SL1	0.90	10.19	0.87	0.10	0.94	28.29	0.92	0.32	0.13	0.91	1.64
SL2	0.89	11.98	0.83	0.14	0.94	33.42	0.89	0.35	0.17	0.95	1.65
SL3	0.54	13.21	0.71	0.44	0.99	76.76	0.93	0.58	0.59	0.94	2.1

## Data Availability

Data are contained within the article; raw data are available upon request.

## Supplementary Materials

The following supporting information can be downloaded at: https://www.mdpi.com/article/10.3390/pharmaceutics18101238/s1, Table S1: Validation parameters of the HPLC method for LUL determination.

## Abbreviations

The following abbreviations are used in this manuscript:

ALG	Sodium alginate
LUL	Luliconazole
PEC	Polyelectrolyte complex
PLL	ε-poly-L-lysine
GEL	Gelatin
FDA	Food and Drug Administration
GRAS	Generally Regarded as Safe
NTU	Nephelometric turbidity unit
TGA	Thermogravimetric analysis
DSC	Differential scanning calorimetry analysis
ATR-FTIR	Attenuated Total Reflectance–Fourier Transform Infrared Spectroscopy
CFU	Colony-forming unit
MTT	3-(4,5-dimethylthiazol-2-yl)-2,5-diphenyltetrazolium bromide
FWMH	Full width at half maximum
