# Supplementary material for: Is It Possible to Perform Triple Cross-Linking of Sodium Alginate? Development of Vaginal Sponges Containing Luliconazole Based on the Alginate/Gelatin/ɛ-Poly-L-Lysine/Zinc Matrix
Source: Pharmaceutics. 2026 Sep 29;18(10):1238. doi: 10.3390/pharmaceutics18101238 (PMC13708505; doi:10.3390/pharmaceutics18101238)
Supplement: Supplementary file 1 [file pharmaceutics-18-01238-s001.zip › pharmaceutics-4573712-supplementary.pdf]

Table S1. Validation parameters of the HPLC method for LUL determination.

Validation parameter	Results
Linearity range	5 – 100 μg/mL
Regression equation	y = 0.513x − 0.1875
Coefficient of determination	R^2 = 0.9999
Accuracy	99.84%
Repeatability	
*RSD**	0.006
*CV***	0.6
Precision	
Intra-day precision	
RSD*	0.01
CV**	1.18
Inter-day precision	
RSD*	0.02
CV**	1.63
Limit of Detection (LOD)	0.67 μg/mL
Limit of Quantification (LOQ)	2.03 μg/mL

*Relative Standard Deviation, **Coefficient of Variation
